# Near-Unity Electrochemical CO_2_ to CO Conversion over Sn-Doped Copper Oxide Nanoparticles

**DOI:** 10.1021/acscatal.2c04279

**Published:** 2022-11-28

**Authors:** Shuang Yang, Zhaochun Liu, Hongyu An, Sven Arnouts, Jim de Ruiter, Floriane Rollier, Sara Bals, Thomas Altantzis, Marta C. Figueiredo, Ivo A.W. Filot, Emiel J.M. Hensen, Bert M. Weckhuysen, Ward van der Stam

**Affiliations:** † Inorganic Chemistry and Catalysis, Institute for Sustainable and Circular Chemistry and Debye Institute for Nanomaterials Science, Utrecht University, Universiteitsweg 99, Utrecht 3584 CG, The Netherlands; ‡ Laboratory of Inorganic Materials and Catalysis, Department of Chemical Engineering and Chemistry, Eindhoven University of Technology, P.O. Box 513, Eindhoven 5600 MB, The Netherlands; § Electron Microscopy for Materials Science (EMAT), University of Antwerp, Antwerp 2020, Belgium; ∥ Applied Electrochemistry and Catalysis (ELCAT), University of Antwerp, 2610 Wilrijk, Belgium

**Keywords:** electrocatalysis, CO_2_ conversion, *in situ* Raman spectroscopy, *in situ* X-ray diffraction, DFT modeling

## Abstract

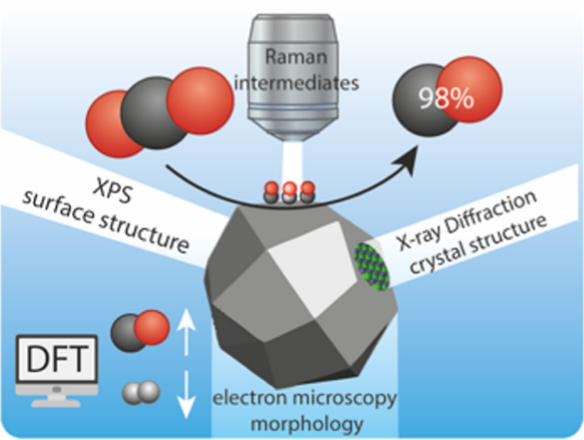

Bimetallic electrocatalysts have emerged as a viable strategy to tune the electrocatalytic CO_2_ reduction reaction (eCO_2_RR) for the selective production of valuable base chemicals and fuels. However, obtaining high product selectivity and catalyst stability remain challenging, which hinders the practical application of eCO_2_RR. In this work, it was found that a small doping concentration of tin (Sn) in copper oxide (CuO) has profound influence on the catalytic performance, boosting the Faradaic efficiency (FE) up to 98% for carbon monoxide (CO) at −0.75 V *versus* RHE, with prolonged stable performance (FE > 90%) for up to 15 h. Through a combination of *ex situ* and *in situ* characterization techniques, the *in situ* activation and reaction mechanism of the electrocatalyst at work was elucidated. *In situ* Raman spectroscopy measurements revealed that the binding energy of the crucial adsorbed *CO intermediate was lowered through Sn doping, thereby favoring gaseous CO desorption. This observation was confirmed by density functional theory, which further indicated that hydrogen adsorption and subsequent hydrogen evolution were hampered on the Sn-doped electrocatalysts, resulting in boosted CO formation. It was found that the pristine electrocatalysts consisted of CuO nanoparticles decorated with SnO_2_ domains, as characterized by *ex situ* high-resolution scanning transmission electron microscopy and X-ray photoelectron spectroscopy measurements. These pristine nanoparticles were subsequently *in situ* converted into a catalytically active bimetallic Sn-doped Cu phase. Our work sheds light on the intimate relationship between the bimetallic structure and catalytic behavior, resulting in stable and selective oxide-derived Sn-doped Cu electrocatalysts.

## Introduction

The electrochemical carbon dioxide (CO_2_) reduction reaction (eCO_2_RR) to produce value-added chemicals and fuels, possibly powered by renewable electricity, has emerged as a promising strategy to valorize CO_2_ emissions from the chemical industry.^1^ The pioneering contributions by Hori *et al.*(69) have inspired numerous researchers over the past decades, and many efforts have been made to develop new catalyst compositions, formulations, and structures with a boosted eCO_2_RR performance. It is generally accepted that eCO_2_RR depends heavily on the catalyst structure and composition and can be influenced by external factors (*e.g.*, composition of the electrolyte).^2,3^ Copper (Cu) stands out as an electrode material because it has displayed the unique capability to reduce CO_2_ to hydrocarbon products, such as ethylene and methane. However, high material cost, low product selectivity, and poor stability remain big challenges that need to be resolved before electrocatalysis can be implemented in industrial applications.^4,5^ Higher production rates and Faradaic efficiencies (FEs) have been reported for two-electron products, such as carbon monoxide (CO), by utilization of silver (Ag)- and gold (Au)-based electrocatalysts. Compared to multi-electron products, such as ethylene (C_2_H_4_) and ethanol (C_2_H_5_OH), which suffer from large overpotentials and limited selectivity, production of two-electron products potentially offers more immediate opportunities for practical application.^6^ For example, CO is one of the main components of syngas (CO + H_2_) used in Fischer–Tropsch synthesis reactions, which generates synthetic petroleum and other fuels.^7^ Therefore, developing advanced and stable electrocatalysts for selective CO generation is of practical importance in the field of electrochemical CO_2_ conversion.

Unfortunately, the electrocatalytically active metals for CO production, such as Ag and Au, require large overpotentials, and their widespread implementation is limited by their high cost.^8,9^ Cu can also catalyze the electrochemical CO_2_ to CO conversion reaction, but due to the intermediate adsorption strength of CO at the Cu surface, these electrocatalysts still suffer from poor selectivity toward CO, and often a wide distribution of reaction products is observed.^10^ It has been reported that the selectivity of Cu for CO and ethylene can be tuned *via* synergistic effects through doping with precious secondary metals, such as Ag and Au.^11,12^ However, it would be beneficial if high selectivity for eCO_2_RR products can be achieved through doping of Cu with cheaper metals to form bimetallic catalysts.

Appealing candidates would be post-transition metals, such as indium (In), lead (Pb), bismuth (Bi), and tin (Sn). Due to their d^10^ electronic configuration, these metals selectively promote CO_2_ to formate conversion and suppress the competing hydrogen evolution reaction (HER).^13,14^ Among these formate-selective metals, Sn-based catalysts have attracted a lot of attention in synergy with Cu due to their solid-state miscibility, the low cost and toxicity of Sn, and the optimized electrocatalytic behavior.^15−21^ Nevertheless, control over stability and selectivity of Cu–Sn bimetallic systems in eCO_2_RR remains a challenge.^22−27^ In addition, although Sn is more abundant and cheaper than Ag and Au, there is still a possibility that Sn could become endangered due to its increasing demand in a variety of manufacturing industries.^28,29^

In this work, we report the synthesis and *in situ* activation of bimetallic Sn-doped Cu electrocatalysts with near-unity CO_2_ to CO conversion for elongated time periods (FE > 90% for 15 h). *Ex situ* characterization through high-resolution high-angle annular dark-field scanning transmission electron microscopy (HAADF-STEM) and X-ray photoelectron spectroscopy (XPS) indicated small SnO_2_ domains at the surface of CuO nanostructures. In the electrochemical CO_2_ conversion measurements, these electrocatalysts showed a boosted selectivity toward CO and required low overpotentials (as low as −0.55 V *vs* RHE) compared to pure CuO electrocatalysts. The maximum FE for CO could reach up to 98% at −0.75 V *versus* RHE for CuO nanoparticles doped with 0.4% Sn, on a par with the most selective CO_2_ to CO conversions reported to date for bimetallic Cu–Sn electrocatalysts (Table S3). *In situ* Raman spectroscopy and X-ray diffraction (XRD) measurements revealed that the pristine Cu–Sn oxide nanoparticles were *in situ* reduced to their metallic phase and that Sn-doping tunes the *CO binding strength. From this, we conclude that the active sites for selective electrochemical CO_2_ to CO conversion are Sn-doped Cu metallic sites. Furthermore, time-resolved *in situ* Raman measurements and density functional theory (DFT) calculations implied that the absorption strength of the main intermediate *CO was weakened due to the Sn doping, facilitating the desorption of CO, resulting in boosted CO formation. Furthermore, DFT calculations revealed that Sn doping weakened the hydrogen adsorption, thereby effectively suppressing the competing HER and boosting CO_2_ to CO conversion to near unity. Our work provides insights into nanoscale synergistic effects in bimetallic Sn-doped Cu electrocatalysts and possibly paves the way to novel design strategies for stable and selective bimetallic Cu-based electrocatalysts.

## Results and Discussion

**Figure 1:**
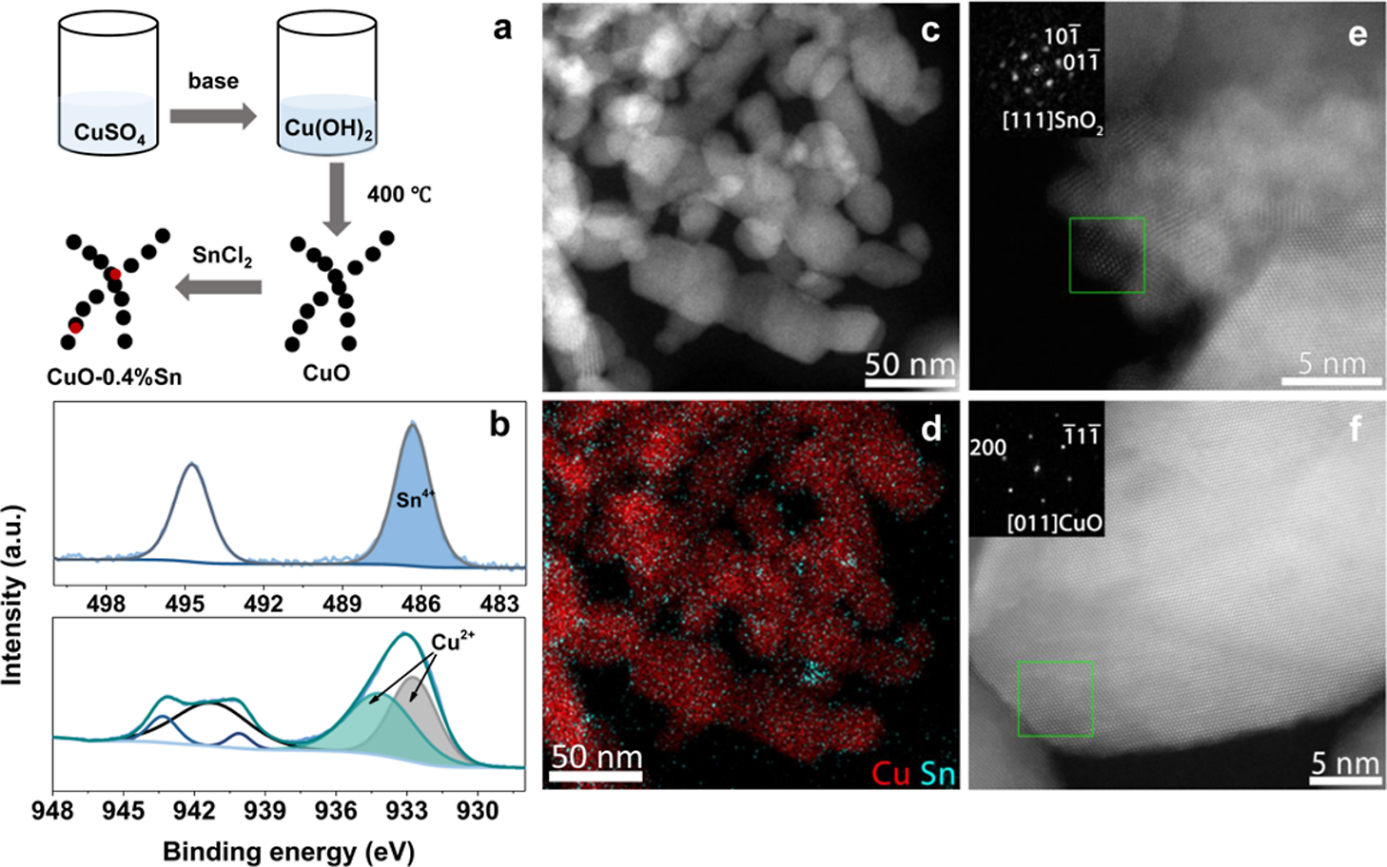
(a) Schematic illustration of the employed catalyst preparation process in this work. (b) XPS measurements of CuO-0.4% Sn for Sn 3d (top) and Cu 2p_3/2_ (bottom). (c) HAADF-STEM overview image of CuO-0.4% Sn and (d) corresponding EDS elemental maps, revealing the distribution of Cu and Sn. (e,f) High-resolution HAADF-STEM images of CuO-0.4% Sn and the corresponding FT patterns (insets). Crystalline domains of SnO_2_ and CuO can be observed in (e,f), respectively.

The pristine CuO catalysts were prepared by a simple wet chemical method (Figure 1a). In this reaction, copper(II) sulfate was dissolved in water, after which ammonia was added to form [Cu(NH_3_)_4_]^2+^ complexes.^30^ The NH_3_ ligands were replaced by OH^–^ through the addition of sodium hydroxide, and the resulting Cu(OH)_2_ nanowires were precipitated (Figure S1). The CuO nanoparticles were obtained through a thermal treatment, in which the Cu(OH)_2_ nanowire precursor decomposed at 400 °C. Afterward, the CuO nanoparticles were doped with tin by dispersing CuO nanoparticles in an ethanol solution containing SnCl_2_ under ultrasonication for 10 min. Sn species formed at the surface of the pristine CuO nanoparticles through a galvanic replacement mechanism.^31^ Varying SnCl_2_ concentrations were used to obtain different degrees of Sn doping, and the Cu/Sn molar ratios were analyzed through inductively coupled plasma-optical emission spectroscopy (ICP-OES) measurements (samples are abbreviated as CuO-*x*% Sn, with *x* = 0.4, 0.6, 0.8, see Table S1).

XPS was further employed to determine the surface chemical state of the different catalyst materials under study. Compared with the bulk Sn/Cu ratios derived from ICP-OES, XPS measurements point to higher Sn/Cu ratios for all Sn-doped samples (Table S2), suggesting that Sn is mainly present at the surface of CuO. The Cu 2p_3/2_ region shows that all catalysts have a main line at 933 eV and a prominent satellite, both characteristic of Cu^2+^ (Figures 1b and S2). From analysis of the Cu 2p_3/2_ region, we can exclude the presence of Cu^0/+^.^32,33^ These observations are confirmed by inspection of the Cu LMM region of the catalysts (Figure S3). In Figures 1b and S4, Sn 3d spectra of all Sn-doped CuO samples evidence the presence of Sn oxides with a main 3d_5/2_ feature at 486.6 eV. Metallic Sn species are not observed.^34,35^ From the Sn 3d spectra, we can derive that Sn is in the oxidized state, but due to the small separation of the corresponding 3d lines, we cannot unambiguously determine the presence of either Sn^2+^ or Sn^4+^. To get more detailed information about the morphological structure and elemental distribution of the pristine and Sn-doped catalysts, the nanoparticles were investigated by transmission electron microscopy (TEM), HAADF-STEM, and coupled STEM energy dispersive X-ray spectroscopy (STEM-EDS) measurements. In Figure 1c, it can be seen that the pristine CuO and Sn-doped CuO nanoparticles have a similar morphology, with an average size of ∼25 nm. This observation already rules out morphology as a possible explanation for variations in catalytic performance, which allows us to selectively study the effect of the dopant on the catalytic behavior.^36^ HAADF-STEM images of pure CuO and the corresponding elemental analysis are given in Figures S6–S8, from which high crystallinity and a uniform distribution of Cu and O can be seen. Figure 1d shows the elemental distribution in the CuO-0.4% Sn sample, where Sn domains can be discerned despite the low Sn content. The Sn domains are located at the surface of the CuO nanoparticles (Figure 1e,f and S9–S11), in line with the XPS results discussed above. The Sn domains are further analyzed with high-resolution HAADF-STEM. From the corresponding Fourier transform (FT) patterns, it can be concluded that Sn doping of CuO nanoparticles resulted in small SnO_2_ domains at the surface of the catalyst, suggesting the Sn^4+^ oxidation state (inset Figure 1e).^37^

The crystalline structure of the prepared pristine samples was determined by X-ray diffraction (XRD) measurements. As shown in Figure S12, no peaks related to crystalline Sn species could be found in the different Sn-doped CuO samples under study, and only the diffraction peaks of CuO without any shifts compared to pure CuO were observed. Together, these findings indicate that Sn is present in a highly dispersed form at the surface of the CuO particles. Furthermore, we conclude that the introduction of Sn did not result in Cu–Sn alloying because this would cause a shift of the characteristic CuO reflections to smaller diffraction angles due to lattice expansion.

**Figure 2:**
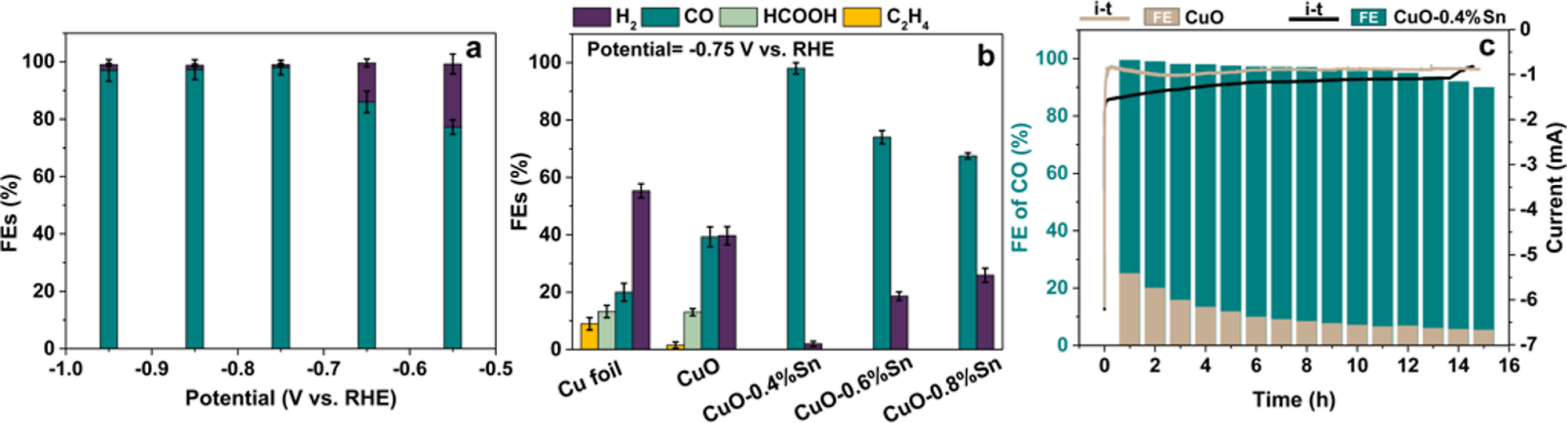
Electrochemical CO_2_ conversion evaluation in CO_2_-saturated 0.1 M KHCO_3_ electrolyte solution. (a) Potential-dependent product distribution of CuO-0.4% Sn. (b) FE comparison of different products on Cu foil, CuO, and Sn-doped CuO at −0.75 V *vs* RHE for 40 min. (c) Long-term chronoamperometry result (line) and the corresponding FE of CO (column) on CuO-0.4% Sn (green) and pristine CuO (light brown) at −0.75 V *vs* RHE for 15 h. The error bars were obtained by performing the experiments in triplicate.

The electrochemical CO_2_ conversion behavior was evaluated in CO_2_-saturated 0.1 M KHCO_3_ solution (pH 6.8) by using a gas-tight H-cell in a standard three-electrode configuration (Ag/AgCl reference electrode, Pt counter electrode, and (Sn-doped) CuO nanoparticles on carbon paper as working electrode). Linear sweep voltammetry (LSV) was employed on the pristine electrocatalysts in a N_2_-saturated electrolyte to analyze the ability of the nanoparticles to catalyze the HER. The pristine CuO nanoparticles displayed the smallest onset potential for HER compared to the Sn-doped CuO catalysts, suggesting a high HER activity, whereas less active HER was observed in the Sn-doped samples (Figure S13). No additional pretreatment of the electrocatalysts was performed prior to eCO_2_RR analysis, which was conducted on freshly prepared electrodes. To analyze the selectivity toward different eCO_2_RR products under various potentials, we applied stepped-potential electrolysis across a potential range from −0.55 to −1.05 V *versus* RHE and detected the gaseous products with online gas chromatography (GC) and liquid products with offline nuclear magnetic resonance (NMR). All Sn-doped CuO samples under study showed boosted selectivity for CO and suppressed HER (<10%), in contrast to pure CuO which produced a wide variety of products and substantial HER (>50%) in the same potential window (Figures 2a and S14). The product distribution of the CuO-*x*% Sn nanoparticles was further compared with Cu foil under the same electrochemical conditions. At an applied potential of −0.75 V *versus* RHE (Figure 2b), the CuO-0.4% Sn sample stood out with a FE of 98% for CO_2_ conversion to CO. This FE is 5-fold and 2-fold higher than that of Cu foil (20%) and pure CuO (40%), respectively.

In addition to the improved CO generation, other common products over Cu-based catalysts, such as ethylene, formic acid, and methane, were effectively inhibited and especially the competitive HER was suppressed. The improvement of CO selectivity clearly depends on the Sn amount. When a higher amount of Sn was introduced into the CuO (CuO-0.6% Sn and CuO-0.8% Sn), HER was only suppressed to a maximum of 20–25% (Figure 2b) and minor hydrocarbon product formation (<3%) was observed at increased cathodic bias (Figure S14). This is probably caused by some bare CuO nanoparticles within the ensemble that remain unaffected by the addition of Sn. The CO enhancement and H_2_ suppression on the investigated Sn-doped CuO samples was further verified by comparing their production rates (Figure S15). Similarly, the most pronounced production rate enhancement was found for CuO-0.4% Sn, while higher amounts of Sn doping led to lower reaction rates. Moreover, a significant difference in the stability test was observed between pure CuO and Sn-doped CuO. As shown in Figures 2c and S16, the Sn-doped CuO samples are stable for CO formation during prolonged electrochemical reduction of CO_2_ (15 h, applied cathodic bias −0.75 V *vs* RHE). The current density also remained stable over the course of hours, whereas a significant change in current density was observed during the *in situ* activation of the CuO–Sn particles to the metallic active phase in the first few minutes (Figure 2c). CuO-0.4% Sn stands out with a FE > 90% CO for 15 h, in contrast to pure CuO nanoparticles which deteriorated over the course of 8 h, after which they only produced half of their initial FE toward CO.

Similar trends can be observed in the intrinsic activity. In Figures S17 and S18, it can be seen that the Sn-doped CuO and pristine CuO samples have similar total geometric current densities. However, the current densities need to be corrected for the electrochemical active surface area (ECSA) due to the enlarged specific surface area caused by nanostructuring of the developed electrocatalysts in this work.^38,39^ In Figures S20 and S21, the small difference in *C*_dl_ for the different samples suggests very similar active surface areas, which is consistent with the trends observed in the total geometric current density analysis. These results imply that morphology is not the critical factor for the boosted catalytic performance of Sn-doped CuO, which is in line with the electron microscopy results described above (Figure 1c–f).

**Figure 3:**
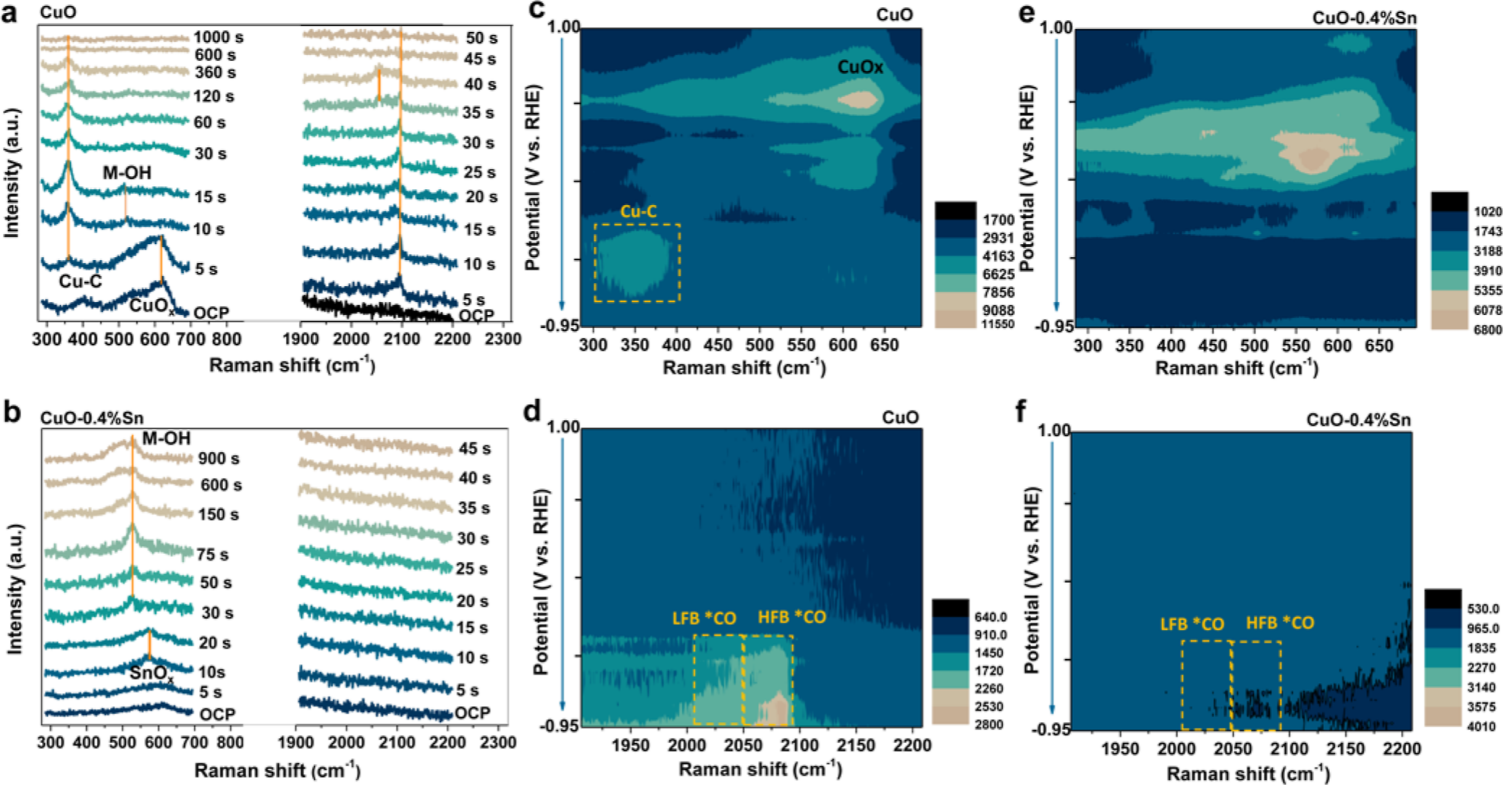
Time-dependent *in situ* Raman spectra of (a) CuO and (b) CuO-0.4% Sn. The measurements were employed at −0.75 V *vs* RHE in a CO_2_-saturated 0.1 M KHCO_3_ electrolyte solution. Potential-dependent *in situ* Raman spectroscopy heatmaps for (c,d) CuO and (e,f) CuO-0.4% Sn. The measurements were employed by scanning the potential from 1.0 V *vs* RHE to −0.95 V *vs* RHE in the CO_2_-saturated 0.1 M KHCO_3_ electrolyte solution (pH = 6.8). The gradient scale bars in (c–f) denote the signal intensity.

To investigate the mechanism behind the CO_2_ to CO promotion in the studied Sn-doped Cu bimetallic systems, *in situ* Raman spectroscopy measurements were conducted in time-dependent and potential-dependent modes (scan speed: 1 spectrum per second). In Figure 3a,b, the time-dependent Raman spectra of CuO and CuO-0.4% Sn are compared at different stages of catalysis at a fixed potential of −0.75 V *versus* RHE. On pure CuO (Figure 3a), surface CuO_*x*_ species are initially present, which are readily reduced at sufficient cathodic bias, evidenced by the disappearance of the CuO_*x*_ Raman signal within 5 s. Subsequently, a Cu–C band starts to appear around 360 cm^–1^, which remains constant for the duration of the experiment.^8,40−42^ This suggests that the pristine CuO nanoparticles are first *in situ* reduced to metallic Cu, which binds CO. Consistent with this, both the low-frequency band (LFB) and high-frequency band (HFB) of adsorbed CO (*CO) are observed as well at 2050 and 2090 cm^–1^, respectively. These LFB and HFB CO bands have been related to C_2_H_4_ formation and CO formation, respectively, in our previous work.^43^ Their coexistence is in line with the catalytic behavior of the CuO nanoparticles, which produce both C_2_H_4_ and CO. Similarly, in CuO-0.4% Sn (Figure 3b), CuO_*x*_ reduction can be seen at the beginning of the reaction, after which SnO_*x*_ (with a characteristic band around 578 cm^–1^) is observed during the first 10 s, which is readily reduced after ∼30 s.^44−48^ The observed band around 578 cm^–1^ is attributed to SnO_*x*_ because many reports have shown that the vibrational modes of SnO_*x*_ species are very complex.^49−51^ The 578 cm^–1^ band could correspond to the S1 mode of SnO_2_ (which would be in line with the observed SnO_2_ islands in the electron microscopy and XPS analysis), but due to the absence of other vibrational modes of SnO_2_, we refer to these bands as SnO_*x*_ hereafter. The SnO_*x*_ bands only become apparent after complete removal of CuO_*x*_ because the CuO_*x*_ vibrations initially dominate over the SnO_*x*_ signal due to the larger CuO_*x*_ volume compared to the SnO_*x*_ islands and the formed metallic Cu enhances the SnO_*x*_ signal through the surface-enhanced Raman spectroscopy (SERS) effect.^52^ The characteristic Cu–C band that was observed for CuO is absent on CuO-0.4% Sn, and a M–OH vibration (with M = Sn or Cu) appears at ∼520 cm^–1^ instead.^53−56^ Vibrational features around 500 cm^–1^ have been debated in the literature,^54^ but often species in this region are attributed to hydroxides generated during catalytic conversion reactions. We attribute the band observed around 500 cm^–1^ to metal-hydroxides, which can be stable at moderate cathodic bias according to the literature.^43,53^ We note that these hydroxide bands are present in both the pristine CuO and Sn-doped CuO samples but that the relative intensity varies due to the presence of dominant Cu–C vibrations in the case of *in situ* activated CuO. In addition to the absence of the Cu–C band, both LFB *CO and HFB *CO cannot be detected for the CuO-0.4% Sn sample. Based on the observations above, we hypothesize that the Cu–C, LFB *CO, and HFB *CO bands become invisible in our Raman spectroscopy measurements due to the fast desorption of surface-absorbed *CO on timescales faster than our time resolution of 1 s. Typically, the observed species in Raman spectroscopy are long-lived species, and detection of short-lived surface intermediates is hampered by the low scattering probability associated with inelastic Raman scattering.^57^

Potential-dependent Raman heatmaps were acquired to gain insights into the dynamics of catalyst activation during cyclic voltammetry scans. Scans were acquired from 1.0 V *versus* RHE to −0.95 V *versus* RHE under *in situ* conditions. As shown in Figure 3c, a similar change in the electrocatalyst structure is found for the pristine CuO nanoparticles during the cathodic scan from 1.0 to −0.95 V *versus* RHE: Initially, CuO is reduced to Cu_2_O, followed by Cu_2_O reduction to metallic Cu.^58^ Reduction to metallic Cu is directly followed by the appearance of the band associated with Cu–C at 360 cm^–1^, evidencing the presence of *CO as confirmed by the spectra in the CO region (Figure 3d, around 2000 cm^–1^). Likewise, we observe LFB *CO and HFB *CO, similar to the time-dependent measurement (Figure 3a). For CuO-0.4% Sn, similar changes are observed in time-dependent and potential-dependent Raman spectroscopy (Figure 3e,f), where bands associated with SnO_x_ (at 578 cm^–1^) appear after copper oxide reduction. These SnO_x_ bands disappear around a potential of 0 V *versus* RHE, indicating full reduction of the Sn-doped CuO nanoparticles to their metallic counterparts. As was found for the time-dependent Raman measurements, no surface bound *CO was present in the potential-dependent measurements. The absence of surface-adsorbed *CO in both time-dependent and potential-dependent Raman spectra suggests that the adsorption strength of intermediate *CO on activated bimetallic Sn-doped Cu nanoparticles is weakened, resulting in enhanced CO generation. Furthermore, we observe a sharp band around 1075 cm^–1^, associated with carbonate ions (CO_3_^2–^), in proximity to the electrocatalyst surface of pristine CuO and CuO-0.4% Sn (Figure S22).^59,60^ The main difference between these samples is that the CO_3_^2–^ signal persists longer on CuO-0.4% Sn than on the pristine CuO, which implies that the adsorbed *CO on the surface is less dominant so that CO_3_^2–^ can approach the electrode for a longer time period.

The samples with different amounts of Sn added (CuO-0.6% Sn and CuO-0.8% Sn) were also investigated with *in situ* Raman spectroscopy under the same conditions. These samples exhibited high CO selectivity as well but also showed some activity for ethylene formation (Figure S14). In their time-dependent *in situ* Raman spectra (Figures S23 and S24), Cu–C and HFB *CO can be clearly discerned, which indicates that the presence of surface absorbed *CO can be attributed to hydrocarbon formation over activated Sn-doped Cu nanoparticles, which requires long-lived *CO intermediates in order to obtain deeper reduction products (*e.g.*, hydrocarbons). Furthermore, these results imply that *CO is more strongly bound to the nanoparticle surface, resulting in lower FE for CO and reactions beyond CO. Their potential-dependent *in situ* Raman heatmaps (Figures S25 and S26) prove the presence of SnO_2_ species and subsequent metal oxide reduction, immediately followed by the appearance of the Cu–C band on these catalysts under cathodic potentials. Moreover, LFB *CO and HFB *CO are found at high overpotentials, in accordance with the observations of CO formation along with a little C_2_H_4_ production at high overpotentials for these catalysts. Similarly, the presence of CO_3_^2–^, which is an indicator for deprotonation close to the surface due to local alkalinity, is observed to persist longer on CuO-0.6% Sn and CuO-0.8% Sn than on pure CuO (Figure S27).

**Figure 4:**
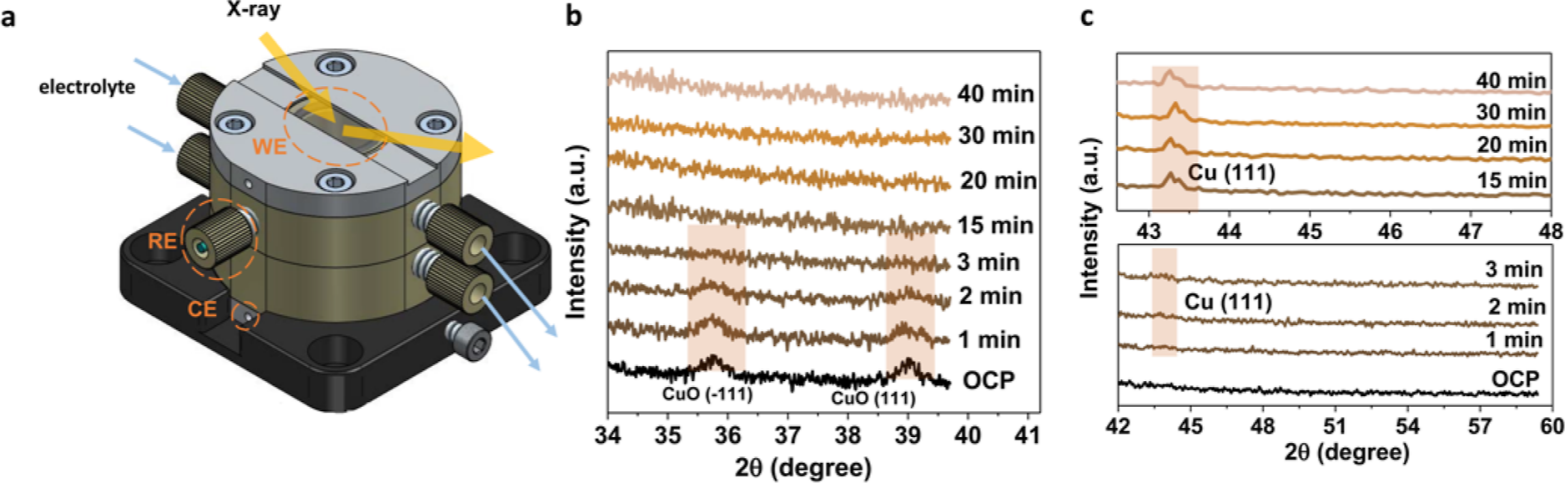
(a) Technical drawing of the *in situ* XRD cell. (b) *In situ* XRD measurements of the CuO-0.4% Sn sample, showing the disappearance of the CuO reflections over time at an applied cathodic bias of −0.75 V *vs* RHE. (c) *In situ* XRD measurements of the CuO-0.4% Sn sample, showing the emergence of the Cu(111) reflection after approximately 3 min of −0.75 V *vs* RHE cathodic bias.

*In situ* XRD measurements were employed to track possible phase changes during catalysis and gain more information about the structure evolution and the activation of the Sn-doped CuO nanoparticles under reaction conditions. For this purpose, an *in situ* XRD cell was designed in house, as depicted in Figure 4a. In this cell, the bulk structure of the active material is probed in a back-illumination mode, ensuring optimal signal to noise and hence time resolution due to suppression of X-ray attenuation caused by water−X-ray interactions. Before the *in situ* investigations, *ex situ* XRD was performed prior to and after catalysis to get an idea about possible structural changes induced by the applied negative potential. As shown in Figure S28, a strong Cu(111) reflection and a small Cu(200) reflection can be observed after catalysis, whereas the reflections of pristine CuO have disappeared. The dynamics of these structural changes were followed by *in situ* XRD measurements, with a time resolution of 1 diffractogram per minute. In the *in situ* measurements (Figure 4b,c), the original CuO(−111) and CuO(111) reflections are observed at open-circuit potential (OCP). These CuO reflections are still vaguely observed after 3 min of −0.75 V *vs* RHE, indicating that full reduction of the CuO nanoparticles is relatively slow, in contrast to the sudden surface oxide reduction observed with Raman spectroscopy (Figure 3). In the diffractogram acquired after 2 min of cathodic bias, a weak reflection is observed at 43.8°, typically associated with metallic Cu(111). This reflection grows in intensity and becomes sharper over time, indicating full reduction of the CuO nanoparticles to metallic Cu and an increase in crystalline domain size, evidenced by a decrease in peak width. These observations are in good agreement with the *in situ* Raman spectroscopy results, which indicated that surface CuO would be reduced within seconds at the onset potential. However, the *in situ* XRD results show that it takes at least 3 min of applied cathodic bias to fully convert the bulk phase of CuO-0.4% Sn to metallic Cu, evidenced by the disappearance of CuO reflections and the emergence of Cu(111). The electrochemical reduction of CuO was confirmed by the HAADF-STEM measurements after catalysis (all samples were tested at −0.75 V *vs* RHE for 40 min, Figures S29–S32). It is observed that both the CuO and the Sn-doped CuO nanoparticles become hollow after catalysis, potentially due to the nanoscale Kirkendall effect (see Figures S29, S30).^61^ The lattice fringes of metallic Cu can be clearly discerned (Figure S31), and a thin CuO layer is also observed on the surface of the Sn-doped CuO catalyst (Figure S32), which can be attributed to the inevitable contact with air during sample transfer. From the STEM- EDS measurements (Figure S33), it can be seen that Sn signal is absent after catalysis. One possible reason is that the elements redistribute during catalysis in the bimetallic system.^62,63^ In this case, the Sn domain of the pristine catalyst dissolved and homogeneously redistributed over the catalyst surface, leading to a lower local Sn concentration that falls below the typical detection limit of the technique (0.1–1 at %). This hypothesis is confirmed by ICP-OES measurements after catalysis (Table S4), which shows that no Sn is leached during catalysis and similar total concentrations of Sn are observed (<1%). Finally, *ex situ* XPS measurements were conducted after catalysis (Supporting Discussion, Figure S34, and Tables S5, S6). It was found that the copper and tin domains were reduced during catalysis but partially reoxidized into Cu(OH)_2_, Cu_2_O, and SnO_*x*_ due to the inevitable air exposure during sample transfer. Furthermore, from the differences in Sn content in the pristine and spent samples, we conclude that the SnO_2_ domains redistribute over the catalyst surface, in line with the observations above: some areas of the catalyst surface are enriched in Sn content, whereas the Sn concentration decreases in other measurement spots. This confirms that the Cu–Sn active phase is formed *in situ* from the pristine CuO–SnO_2_ electrocatalyst and that Sn is redistributed over the surface.

The *in situ* Raman spectroscopy and *in situ* XRD (Figure S35) results have indicated that the pristine Sn-doped copper oxide nanoparticles are activated *in situ* and form metallic Cu and Sn active sites for selective CO_2_ conversion to CO. By comparison of the activity of undoped and doped nanoparticles and our *in situ* investigations, it becomes evident that Sn doping tunes the adsorption strength of intermediate *CO on the Cu-based catalyst surface. This is evidenced by the *in situ* Raman spectroscopy measurements, in which sharp Cu–C bands and surface adsorbed *CO are discerned on CuO, whereas *CO is not observed when CO formation is close to unity over CuO-0.4% Sn. It is likely that the desorption of CO on activated Sn-doped Cu nanoparticles is too fast to be detected with the time resolution of our *in situ* Raman spectroscopy measurements (1 s).

**Figure 5:**
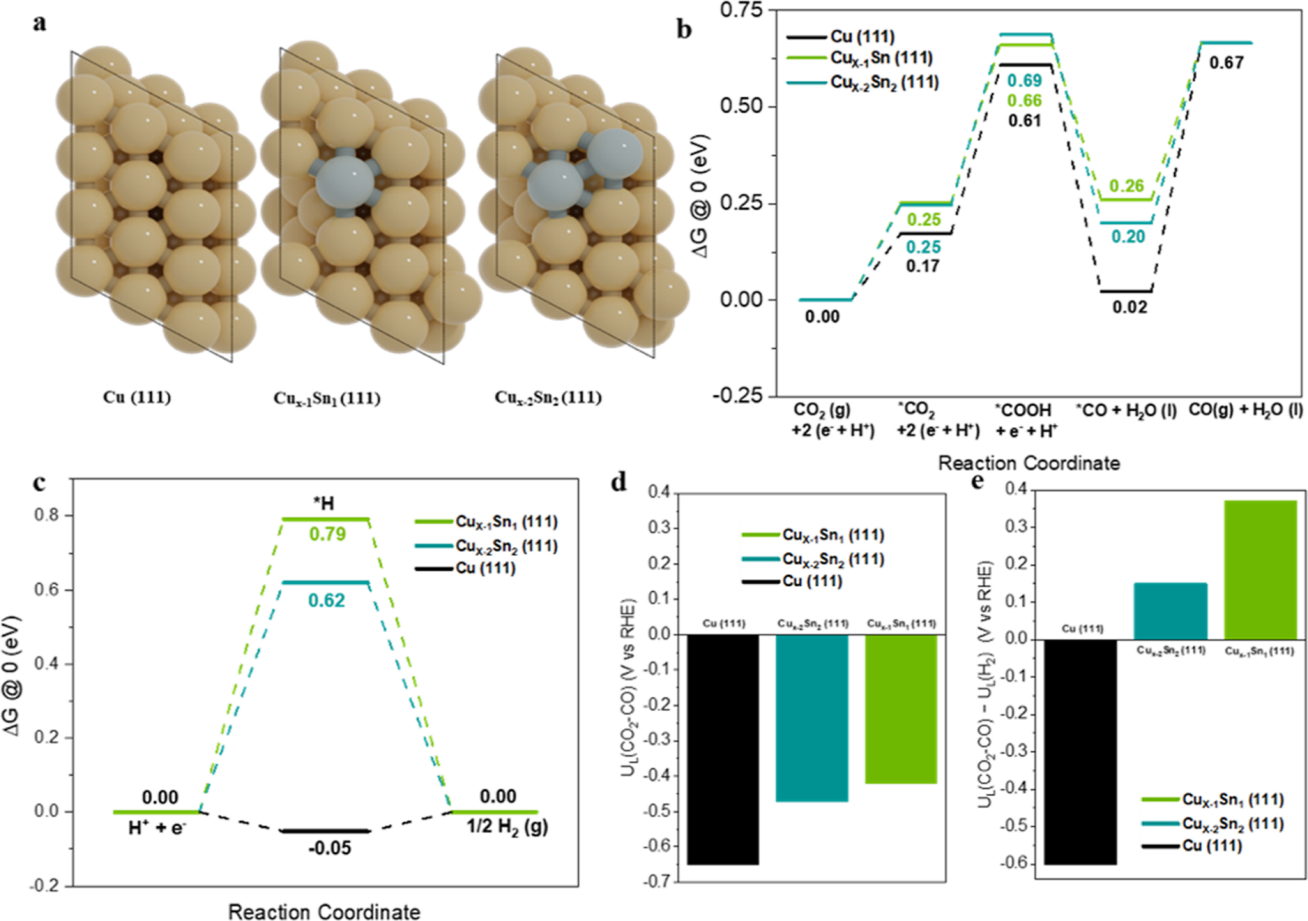
DFT calculations of the eCO_2_RR and HER on CuSn and Cu surfaces. (a) Top views of model 111 slabs for Cu, Cu_*X*–1_Sn, and Cu_*X*–2_Sn_2_. Gibbs free-energy (Δ*G*) diagrams for (b) CO_2_ reduction to CO on model systems and (c) H_2_ evolution on model systems. (d) Computed limiting potentials for CO_2_ reduction. (e) Difference in limiting potentials for CO_2_ reduction and H_2_ evolution.

In order to understand possible mechanistic differences between the reduced CuO and Sn-doped CuO electrocatalysts, DFT calculations were conducted within the computational hydrogen electrode approach developed by Nørskov’s group, while additionally taking into account the solvent implicitly.^64,65^ As structural models, Cu(111), Cu_x–1_Sn(111), and Cu_x–2_Sn_2_(111) were used, in which Sn-substituted Cu in the surface Cu layer, representing the active phases of Cu electrodes at varying Sn-dopant levels (Figures 5a and S36–S38 for detailed information on models and methods used). These structural models are in line with the experimental observations, which showed dominant Cu(111) surfaces with <1% Sn doping. Two Cu/Sn ratios were selected for the theoretical models (Cu/Sn 48:1 and 24:1), which roughly correspond to the atomic ratios at the surface for experimental samples CuO:0.4%Sn and CuO:0.8% Sn, as observed with XPS (2 and 4%, respectively, see Tables S2, S5, S6). The distribution of the Sn dopants in the models was investigated, and the influence of Sn distribution was found to be minimal in the Cu_*x*–2_Sn_2_(111) models (Table S7). Using DFT, Gibbs free energies were computed for the key steps involved in electrochemical reduction of CO_2_ to CO and the competing HER (Figure 5b,c). According to Figure 5b, the Gibbs free energies for the formation of *COOH on Cu_x–1_Sn(111), Cu_x–2_Sn_2_(111), and Cu(111) are, respectively, 0.66, 0.69, and 0.61 eV, indicating that Sn doping does not significantly affect the initial protonation step of CO_2_. According to the computed energy diagrams (Figure 5b), the desorption of *CO to CO(g) is favored on the Sn-doped models: the calculated desorption energies of *CO are 0.41, 0.47, and 0.65 eV for the Cu_x–1_Sn(111), Cu_x–2_Sn_2_(111), and Cu(111) facets, respectively. These differences correlate well with the experimentally observed differences in the CO formation rate, where the Sn-doped CuO nanoparticles displayed higher partial current densities for CO production than CuO nanoparticles.

As the HER is the main reaction that competes with CO_2_ reduction, the Gibbs free energy for *H formation was also computed for the studied surface models (Figure 5c). The corresponding values for Cu_x–1_Sn(111) and Cu_x–2_Sn_2_(111) are found to be 0.79 and 0.62 eV, respectively, which are significantly higher than that for Cu(111) (−0.05 eV). The substantial destabilization of *H on the Sn-doped Cu models with respect to Cu(111) can explain the experimentally observed suppressed hydrogen formation for the bimetallic Sn-doped Cu nanoparticle electrodes.

It is also worthwhile to compare the Raman spectroscopy data to computed CO adsorption on Cu sites in the models. It has been earlier established that CO adsorption on Sn is very weak.^66^ The data in Figure S39 shows that CO adsorption by Cu_x–1_Sn(111) and Cu_x–2_Sn_2_(111) is weaker than by Cu(111). Notably, the experimental data suggests that CO is weakly adsorbed on the catalytic surface of the Sn-doped CuO nanoparticles. This may be interpreted as a significant part of the Cu surface being terminated by Sn sites because either no Cu ensembles are available or these sites bind CO weakly because of the nearby presence of Sn. Thus, the presence of Sn at the surface of the *in situ* activated electrocatalyst tunes the adsorption strength of the key intermediate *CO.

**Scheme 1:**
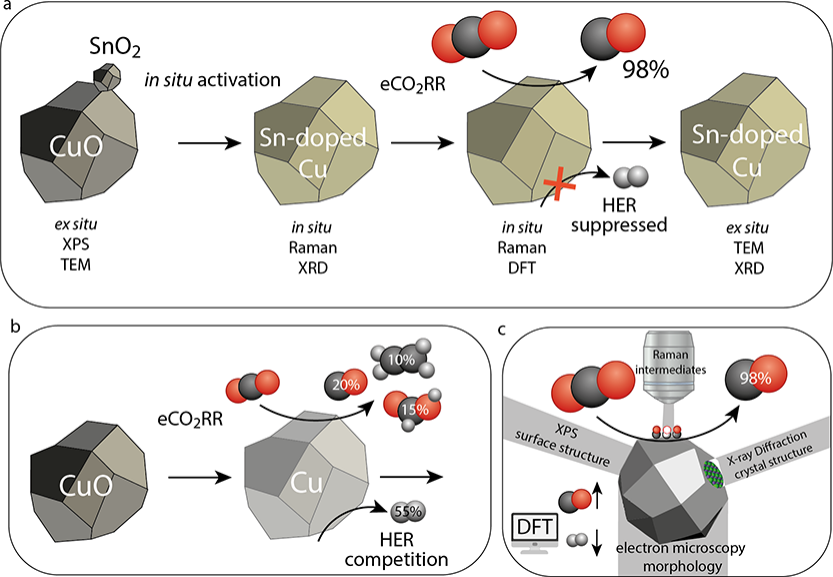
Summary of the *In Situ* Activation and Electrocatalytic CO_2_ Reduction Performance of (a) Sn-Doped CuO Nanoparticles and (b) Pristine CuO Nanoparticles, as Evidenced by (c) Combination of *Ex Situ* and *In Situ* Techniques and DFT Calculations

These results can also be discussed in terms of the limiting potential *U*_L_, which is the potential at which a reaction step becomes exergonic. The limiting potential *U*_L_ equals −Δ*G*/e with Δ*G* being the Gibbs free-energy change for the potential-limiting step. As the activation barriers scale with such Gibbs free reaction energies, trends in *U*_L_ follow trends in activity.^67^Figure 5d shows the thermodynamic limiting potentials *U*_L_(CO_2_–CO) for the three model systems. *U*_L_(CO_2_–CO) is the most positive for Cu_*x*–1_Sn(111) followed by Cu_*x*–2_Sn_2_ (111) and Cu(111), consistent with the order in overall current densities observed in the experiments. The difference between the limiting potentials for CO_2_ reduction and H_2_ evolution, that is, *U*_L_(CO_2_–CO) – *U*_L_(H_2_), reflects the difference in reaction rates toward CO and H_2_ and, henceforth, the CO selectivity.^68^ As shown in Figure 5e, the computed differences in *U*_L_(CO_2_–CO) – *U*_L_(H_2_) can explain well the decrease in FE to CO when going from CuO-0.4% Sn to CuO-0.8% Sn and CuO. Overall, the DFT calculations help to understand why CuO-0.4% Sn is the catalyst with the highest electrocatalytic activity and increased CO selectivity due to a lower binding energy of CO and H. Scheme 1 summarizes the *in situ* activation of the pristine electrocatalysts and the differences in performance between pure CuO and Sn-doped CuO electrocatalysts, as analyzed by a combination of *ex situ* and *in situ* characterization techniques and DFT calculations.

## Conclusions

In this work, Sn-doped Cu bimetallic electrocatalysts are activated *in situ* from pristine SnO_2_-decorated CuO nanoparticles, which displayed near-unity selectivity for CO_2_ to CO conversion with stable performance for up to 15 h. The pristine morphology and structure were elucidated through *ex situ* electron microscopy, X-ray spectroscopy, and diffraction measurements, which revealed that CuO nanoparticles were decorated with SnO_2_ domains. The *in situ* activated Sn-doped Cu electrocatalysts displayed improved electrochemical CO_2_ conversion to CO, with a record FE of 98% for CuO-0.4% Sn at an applied potential of −0.75 V *versus* RHE. Time- and potential-dependent *in situ* Raman spectroscopy and *in situ* XRD measurements were utilized to reveal the activation of the catalyst and the adsorbed species at the catalyst surface. We find that pristine surface CuO is readily reduced within a few seconds, resulting in the presence of *CO and Cu–C vibrations in the Raman spectra, whereas full reduction of bulk CuO takes at least 3 min according to the *in situ* XRD measurements. Sn-doping resulted in the absence of *CO vibrations in the Raman spectra, suggesting fast desorption of gaseous CO, in line with the near-unity electrocatalytic performance. This was confirmed by DFT calculations, which revealed that the secondary component (Sn) strongly suppresses the HER and weakens the adsorption strength of the key intermediate *CO on the catalyst surface, leading to boosted CO generation. This work opens up an attractive avenue to develop high-performance and low-cost electrocatalysts through doping of Cu-based electrocatalysts with post-transition metals and provides insights into nanoscale synergistic events and *in situ* activation of oxide-derived Sn-doped Cu nanoparticles.

## Supporting Information Available

Synthetic procedures, structural characterization (XRD, ICP-OES, XPS, TEM, HAADF-STEM, and STEM-EDX) of pristine and spent samples, *in situ* Raman spectroscopy, computational methodology, and catalytic performance analysis (PDF)
