# Supplementary material for: Near-Unity Electrochemical CO2 to CO Conversion over Sn-Doped Copper Oxide Nanoparticles
Source: ACS Catal. 2022 Nov 28;12(24):15146–56. doi: 10.1021/acscatal.2c04279 (PMC9764354; doi:10.1021/acscatal.2c04279)
Supplement: Supplementary file 1 — cs2c04279_si_001.pdf [file cs2c04279_si_001.pdf]

Supporting Information

Near Unity Electrochemical CO_2 to CO Conversion over Sn-doped Copper Oxide Nanoparticles

Shuang Yang,[†] Zhaochun Liu,[‡] Hongyu An,[†] Sven Arnouts,[§,ʃ] Jim de Ruiter,[†] Floriane Rollier,[‡] Sara Bals,[§] Thomas Altantzis,[ʃ] Marta C. Figueiredo,[‡] Ivo A.W. Filot,[‡] Emiel J.M. Hensen,[‡] Bert M. Weckhuysen[,†] and Ward van der Stam*[,†]*

[†] Inorganic Chemistry and Catalysis, Institute for Sustainable and Circular Chemistry and Debye Institute for Nanomaterials Science, Utrecht University, Universiteitsweg 99, 3584 CG Utrecht, The Netherlands.

[‡] Laboratory of Inorganic Materials and Catalysis, Department of Chemical Engineering and Chemistry, Eindhoven University of Technology, P.O. Box 513, 5600 MB Eindhoven, The Netherlands.

[§] Electron Microscopy for Materials Science (EMAT), University of Antwerp, 2020 Antwerp, Belgium.

[ʃ] Applied Electrochemistry and Catalysis (ELCAT), University of Antwerp, 2610 Wilrijk, Belgium.

Email: b.m.weckhuysen@uu.nl and w.vanderstam@uu.nl

A. Experimental Section

Catalyst preparation

CuO nanoparticles were synthesized by a wet chemical method, modified from a previously reported work.[1] First, 998 mg $CuSO_4.5H_2O$ was dissolved in 100 mL ultrapure water, followed by the addition of 30 mL 0.15 M $NH_3.H_2O$ solution. After stirring for 15 min, 6 mL 1.2 M NaOH solution was added into the above solution dropwise, followed by stirring for another 20 min at room temperature. Subsequently, the obtained suspension containing blue sediment was separated by filtration. The sample collected from filtration was dried in the air at room temperature. When the samples were fully dried, the powder was annealed in an air furnace at 400 ℃ for 2 h, with a heating rate of 1 ℃ min^{-1}. The preparation of Sn doped CuO was achieved through galvanic replacement. 10 mg, 20 mg and 30 mg of $SnCl_2$ was dispersed in 40 ml absolute ethanol under ultrasonication for 5 min, followed by the addition of 30 mg CuO nanoparticles, to form CuO-0.4%Sn, CuO-0.6%Sn and CuO-0.8%Sn, respectively. After ultrasonication for another 5 min, the samples were collected and washed with a mixture of ultrapure water and ethanol and dried in the air overnight.

Catalyst Characterization

The elemental composition of the pristine samples was characterized by Inductively Coupled Plasma-Optical Emission Spectrometry (ICP-OES, PerkinElmer Optima 8300 Optical Emission Spectrometer). An average of three samples was used. The phase structure of studied materials was determined by X-Ray Diffraction (XRD) measurements on a Bruker D2 Phaser diffractometer for *ex situ* measurement and Bruker D8 Phaser diffractometer for *in situ* measurement, equipped with a Co Kα source (λ = 1.78896 Å) and a Cu Kα source (λ = 1.54056 Å), respectively. The morphology and elemental distribution were investigated by Transmission Electron Microscopy (TEM, Tecnai20, 200 keV) and High Angle Annular Dark Field Scanning Transmission Electron Microscopy (HAADF-STEM) and coupled STEM Energy Dispersive X-ray Spectroscopy (STEM-EDS) measurements (ThermoFischer Scientific Titan, equipped with the ChemiSTEM system, 200 keV). To do this with the catalyst after catalysis, the particles were scraped of the carbon paper with the help of a scalpel, dispersed in EtOH and dropcasted on the TEM grid. In the X-ray Photoelectron Spectroscopy (XPS) measurements, a K-Alpha X-ray photoelectron spectrometer by ThermoFisher scientific with an aluminum (K= 1486.68 eV) X-ray source was used to collect the X-ray photoelectron spectra. All spectra were calibrated with reference to the C 1s at 284.5 eV. The curve fitting was carried out using Shirley background and Gaussian function.

Electrochemical Measurements

The Ivium compactstat.h10800 potentiostat was used for electrochemical performance measurements. In a gas-tight H-cell with two separated chambers, a standard three-electrode system was built with a proton exchange membrane (Nafion 117, Dupont). An Ag/AgCl (ET069-1, −0.205 V vs. SHE) and a Pt-mesh were employed as reference electrode and counter electrode, respectively. Before starting measurement, 0.1 M $KHCO_3$ aqueous solution was saturated with CO_2 for at least 30 min to obtain an electrolyte with pH of 6.8. During the experiments, CO_2 was continuously delivered into the cathodic chamber at a constant rate of 10 mL min^{-1}. To prepare working electrodes, 5 mg catalyst powder was mixed with 20 µL of Nafion solution (5 wt.%) and 500 µL methanol to form catalyst ink after ultrasonication for 2 h. The working electrode was fabricated by drop casting the catalyst ink onto carbon paper (effective electrode area 2 cm^2). The Electrochemical Surface Area (ECSA) was evaluated by the double-layer capacitance (C_{dl}) through the Cyclic Voltammetry (CV) measurements at different scan rates in the non-Faradaic region by a linear fit of the charging current. The solution resistance (Rs) was determined by Electrochemical Impedance Spectroscopy (EIS) with a frequency range of 10 Hz to 100 kHz for the *iR* correction. The measured potential values were converted to the Reversible Hydrogen Electrode (RHE) using the following equation.

$$\mathrm{E\ (vs.RHE)} = \mathrm{E\ (vs.Ag/AgCl)}\ +\ 0.205\ \mathrm{V}\ +\ 0.059 \times \mathrm{pH}\ - iR$$

The potential-dependent catalytic performance was probed by potentiostatic test at different cathodic potentials. Each potential was held for at least 40 min.

Products Analysis

In this work, an online Gas Chromatography (GC) was used for gas products analysis, which is equipped with a Thermal Conductivity Detector (TCD) and a Flame Ionization Detector (FID). For liquid products determination, ^{1}H Nuclear Magnetic Resonance (NMR) spectroscopy was employed using water suppression mode. After catalysis, 0.5 mL of electrolyte was extracted and mixed with 0.1 mL of deuterated water (D_2O) as lock solvent. 0.05 µL of dimethyl sulfoxide (DMSO) was added as the internal reference.

The Faradaic Efficiency (FE) of gas products was calculated using the following equation.

$$FE = \frac{n\ .\ F.\ c\ .\ f}{Vm\ .\ I\ .\frac{60sec}{min}.\ 1000000ppm} \times 100\%$$

Production rate of all products were calculated by using the following equation:

$$production\ rate = \frac{FE \cdot I}{n \cdot F \cdot S}$$

where *n* represents the number of electrons involved to produce the related products from CO_2 or H_2O (e.*g.*, 2 for CO and H_2, 12 for C_2H_4); *F* is the Faraday constant (96485 C mol^{-1}); *c* is the concentration of the product measured by GC (ppm); *f* is the gas flow rate (mL min^{-1}); *I* is the average measured current in 1 min (A); *Vm* is the volume of 1 mol gas at reaction temperature and pressure (24451 mL mol^{-1}); S represents the geometric area of the electrode (cm^2).

The FE of the liquid products was calculated by using the following equation.

$$FE = \frac{n\ .\ F\ .\ M\ .\ V}{I.\ t} \times 100\%$$

where *n* is the number of electrons transferred to form the desired product (e.*g.*, 2 for HCOOH); *F* is the Faraday constant (96485 C mol^{-1}); *M* is the molar concentration of the liquid product; *V* is the liquid volume; *I* is the average measured current in 1 minute (A); *t* is the duration time (s).

In situ Raman Spectroscopy Measurements

A Renishaw InVia Raman microscope and 785 nm excitation laser were used for the *in situ* Raman spectroscopy measurements, coupled with a Nikon N40X-NIR water-dipping objective. The laser power was set to below 1.5 mW in order to avoid laser damage. The time interval for each spectrum is 1 s for all measured samples. The *in situ* Raman spectroscopy measurements were coupled with CV, for which an Autolab PGSTAT 101 potentiostat was used with a scanning rate of 50 mV s^{-1} in CO_2 saturated 0.1 M $KHCO_3$ electrolyte solution (pH 6.8), from 1.0 V to -0.95 V vs. RHE.

In situ XRD Measurements

A Bruker D8 Phaser diffractometer was employed for the *in situ* XRD measurements. In a custom-made *in situ* cell, a glassy carbon wafer (SIGRADUR K films, diameter 22 mm, thickness 180 µm) coated with catalysts, a Pt wafer and an Ag/AgCl electrode were used as working electrode, counter electrode and reference electrode, respectively. Back-illumination configuration was used in the measurement under Bragg mode. The electrolyte (i.e., a CO_2 saturated 0.1 M $KHCO_3$ solution) was introduced into the cell with a flow rate of 10 mL/min. The Ivium compactstat.h10800 potentiostat was used for the potentiostatic test with a potential fixed at -0.75 V vs. RHE for 40 min.

Density Functional Theory calculations

Spin-polarized Density Functional Theory (DFT) calculations were performed with the Vienna Ab Initio Simulation Package (VASP 5.4.1).[2] The ion-electron interactions are represented by the projector-augmented wave (PAW) method[3] and the electron exchange-correlation by the generalized gradient approximation (GGA) with the Perdew-Burke-Ernzerhof (PBE) functional.[4] An energy cut-off of 400 eV was employed for the plane-wave basis set. The convergence threshold for ionic steps in geometry optimization was 1×10^{-5} eV. Geometry optimization was deemed converged when the forces on each atom were below 0.02 eV/Å. The geometry optimization was followed by a frequency analysis to make sure that the optimized structures are genuine minima. The Van der Waals interactions determined via the DFT-D3 method were considered in all of our calculations.[5]

For the modeling of the CuSn surfaces, the models were a periodic ceria slab with a (4×3) surface supercell containing four layers, in which the bottom two layers were frozen. According to the atomic ratios shown in Table S2 by the XPS measurements results, Cu (111) surface with 48 Cu atoms was constructed, one or two of the Cu atoms at the top layer of the surface were replaced by Sn, describing the structure of $Cu_{x-1}Sn_1$, and $Cu_{x-2}Sn_2$, respectively. Neighboring slabs were separated by a vacuum of 20 Å to avoid self-interactions. Brillouin zone sampling was restricted to the Γ-point for the clusters and Monkhorst-Pack k-point meshes of 5 × 3 × 1 were used for the periodic surfaces.

For implicit solvation calculations, we have employed the VASPsol[6] software package that incorporates the solvation effect into VASP within a self-consistent continuum model. VASPsol has been generally applied in different electrochemical systems recently due to its simplicity and low computational costs. The energy from DFT is added to the energies from electrostatic interactions between the solute and the solvent and the cavitation energy to create the solute within the solvent. The adopted solvent is water with a dielectric constant of 78.4.

Thermodynamic properties were estimated by means of in-house software LiuZCpy. The Gibbs free energies at 298.15 K and 1 atm were calculated with the following equation:

$$\Delta G = \Delta E_{DFT} + \Delta E_{ZPE} + \Delta G_U + \Delta G_{PH} + \Delta\int_0^{298.15K} C_P dT - T\Delta S$$

where ΔE_{DFT} is the adsorption energy of CO_2RR intermediates, ΔE_{ZPE} is their zero-point vibrational energy, C_P is the constant-pressure heat capacity, , ΔG_U= -eU, where e is the elementary charge, U is the electrode potential and is set here as 0V. ΔG_{PH} is the correction of the H^+ free energy by the concentration, $\Delta G_{pH} = 2.303 \times k_{BT} \times$ pH (or 0.059 × pH), here the value of pH was assumed to be zero. ΔS is the entropy change at finite temperature T and the integration terms are calculated based on the vibrational energies of CO_2RR intermediates. The entropies of free gas-phase molecules were obtained from the NIST-JANAF thermochemical tables (https://janaf.nist.gov/). The computational hydrogen electrode (CHE) model[7] was used to calculate the free energy of electro-catalytic CO_2 reduction.

Adsorption energies of the CO intermediate was computed by using the following equation:

$$\Delta G_{ads}(CO) = G(facet\text{-}CO) - G(facet) - G(CO)$$

where G(facet-CO), G(facet), and G(CO) are are the free energies of the specie adsorbed on the surface G(facet-CO), the empty surface G(facet) and the corresponding gas phase specie (CO), respectively.

B. Supplementary Tables

Table S1. Overview of the ICP-OES results of molar percentage of Sn in the different Sn doped CuO samples under study.

Samples	Cu (mol%)		Sn (mol%)	
CuO-	99.59		0.41	
0.4%Sn	99.55	99.60 (±0.05)	0.45	0.40 (±0.05)
	99.60		0.40	
CuO-	99.40		0.60	
0.6%Sn	99.39	99.40 (±0.05)	0.61	0.60 (±0.04)
	99.44		0.56	
CuO-	99.25		0.75	
0.8%Sn	99.21	99.20 (±0.05)	0.79	0.80 (±0.05)
	99.18		0.82	

Table S2. Overview of the XPS results of atomic percentage of Cu and Sn in the different Sn doped CuO samples under study.

Samples	Cu (at.%)	Sn (at.%)
CuO-0.4%Sn	98.206	1.794
	98.209	1.791
CuO-0.6%Sn	97.597	2.403
	97.578	2.422
CuO-0.8%Sn	95.923	4.077
	96.123	3.877

Table S3. Summary of the Cu-Sn bimetallic electrocatalysts for CO_2 conversion to CO and HCOOH.

	Synthesis	Electrolyte	Potential V vs. RHE	FE of CO	FE of HCOOH	References
$Cu_{6.26}Sn_5$	Hydrogen bubble templates	0.1 M $KHCO_3$	−1.08	-	97.80%	*Appl. Catal. B: Environmental* ***2021****, 292, 120119*
SnO_2/CuO NCs	Co-precipitation	0.5 M KHCO3	−1.00	-	89.30%	*ChemElectroChem* ***2021****, 8, 1150– 1155*
Hollow Cu/Sn	Colloidal synthesis	0.1 M $KHCO_3$	−1.00	20.10%	70.10%	*Nat Commun* ***2018****, 9, 4933*
Cu/p-Sn	Physical vapor deposition	0.1 M $KHCO_3$	−1.00	58.10%	24.10%	*ACS Appl. Energy Mater.* ***2020****, 3, 11, 10568–10577*
CuNW-Sn15c	Atomic layer deposition	0.1 M $KHCO_3$	−0.70	79.00%	6.70%	*Adv. Energy Mater.* ***2022****, 12, 2103328*
Cu_3Sn/Cu	Alloying/dealloying	0.1 M $KHCO_3$	−0.70	91.50%	-	*Small* ***2021****, 17, 2100683*
$Cu_{20}Sn_1$	one-step reduction	0.5 M $KHCO_3$	−1.00	95.30%	-	*ACS Catal.* ***2021****, 11, 17, 11103–11108*
$Cu_{97}Sn_3$	one-step reduction	0.5 M $KHCO_3$	−0.70	98.00%	-	*Nat Commun* ***2021****, 12, 1449*
CuO-0.4%Sn	Galvanic replacement	0.1 M $KHCO_3$	−0.75	98.00%	-	This work

Table S4. Overview of the ICP-OES results of molar percentage of Sn in the different Sn doped CuO samples after catalysis.

Samples	Cu (mol%)	Sn (mol%)
CuO-0.4%Sn	99.57	0.43
CuO-0.6%Sn	99.41	0.59
CuO-0.8%Sn	99.19	0.81

Table S5. Overview of the XPS results of atomic percentage of Cu and Sn in the different Sn doped CuO samples after catalysis at −0.75 V vs. RHE for 40 min.

Samples	Cu (at.%)	Sn (at.%)
CuO-0.4%Sn	98.424	1.576
CuO-0.6%Sn	98.709	1.291
	97.422	2.578
CuO-0.8%Sn	98.293	1.707
	97.760	2.240

Table S6. Overview of the XPS results of atomic percentage of $Cu(OH)_2$ and Cu_2O from the analysis of the Cu2p3/2 lines after catalysis at −0.75 V vs. RHE for 40 min.

**Sample**	**$Cu(OH)_2$**	**Cu_2O**
CuO spent spot1<br>spot2	67.79%<br>59.7%	32.21%<br>40.3%
CuO – 0.4% Sn spent spot1	63.18%	36.82%
CuO – 0.6% Sn spent spot1<br>spot2	70.19%<br>58.07%	29.81%<br>41.93%
CuO – 0.8% Sn spent spot1<br>Spot2	61.15%<br>53.66%	38.85%<br>46.34%

Table S7. Relative energy in Sn atoms doped Cu (111) surface.

dopingPosition	$Cu_{x-1}Sn$ Relative energy/eV	dopingPosition	$Cu_{x-2}Sn_2$ Relative energy/eV
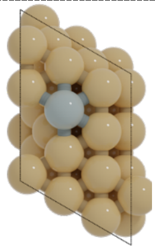	0	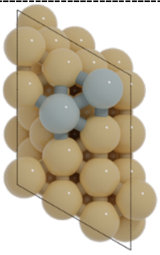	0
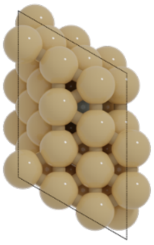	1.49	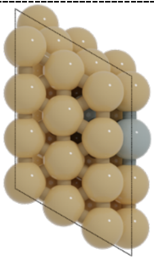	2.95

C. Supplementary Figures

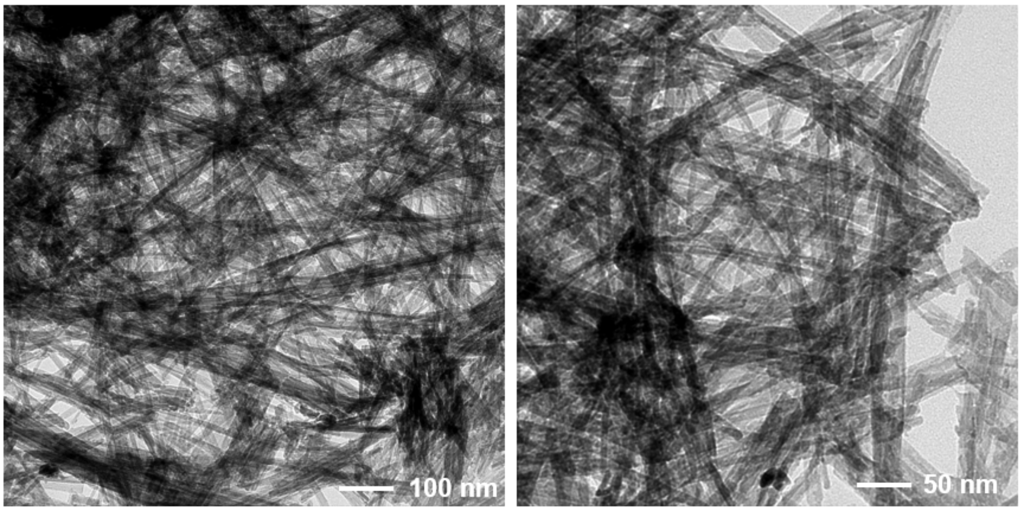

Figure S1. TEM images of the $Cu(OH)_2$ nanowire precursor measured with different magnifications.

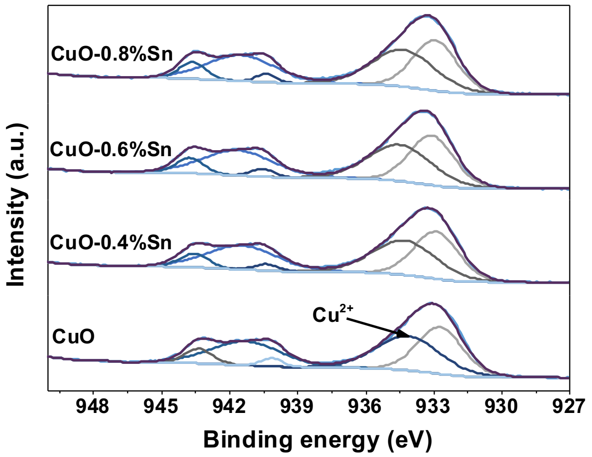

Figure S2. Cu 2p XPS data of CuO and the different Sn doped CuO materials under study.

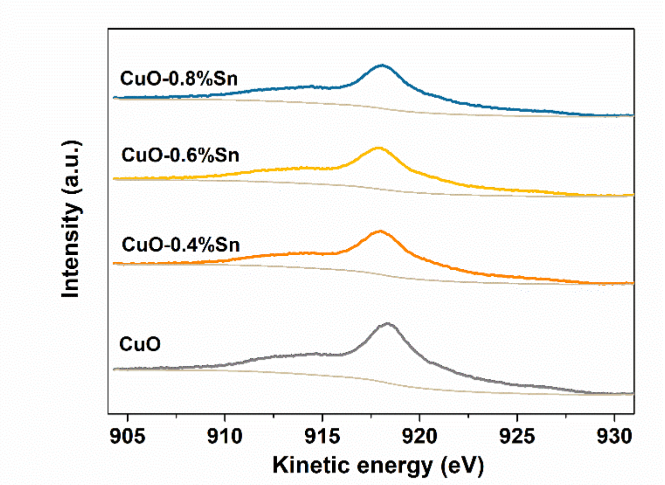

Figure S3. Cu LMM data of CuO and the different Sn doped CuO materials under study. The peak located at 918.10 eV can be attributed to surface Cu^{2+} species.[8]

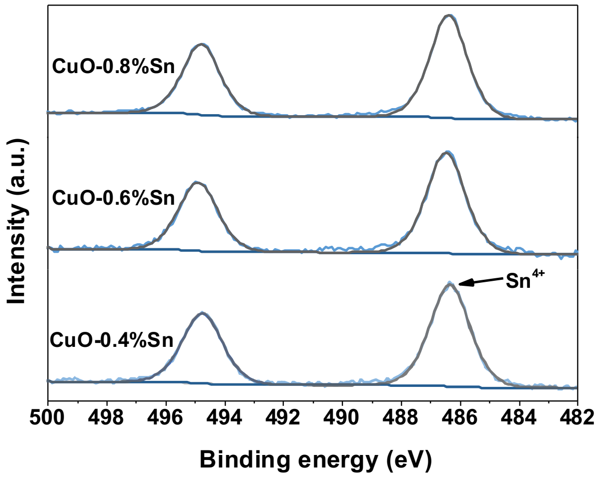

Figure S4. Sn 3d XPS data of the different Sn doped CuO materials under study.

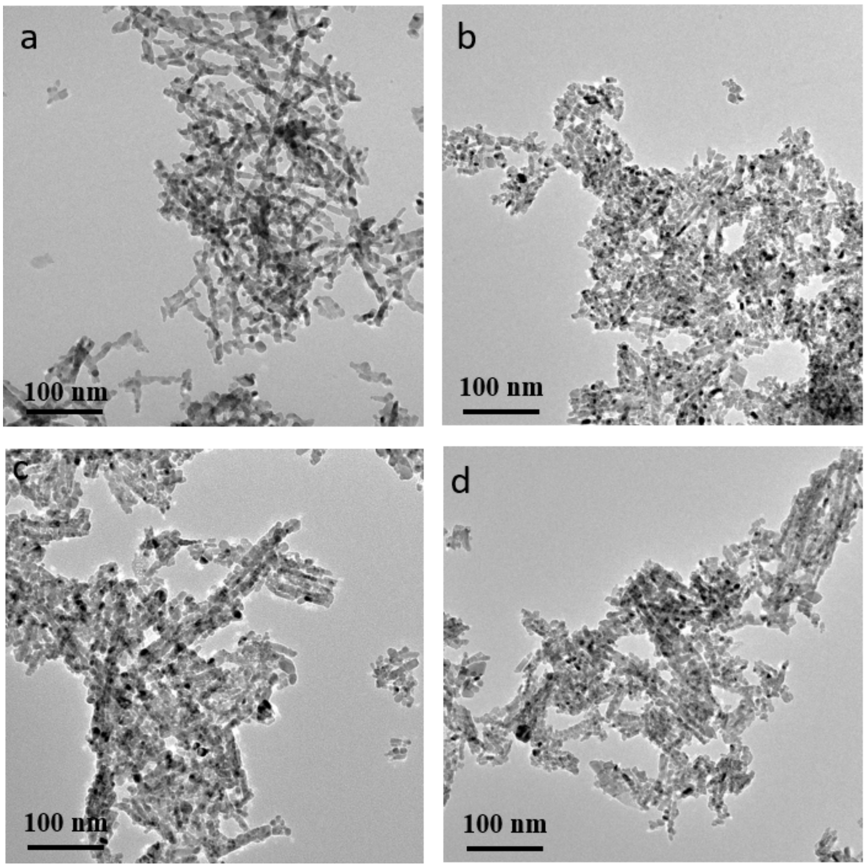

Figure S5. TEM images of (a) CuO, (b) CuO-0.4%Sn, (c) CuO-0.6%Sn and (d) CuO-0.8%Sn.

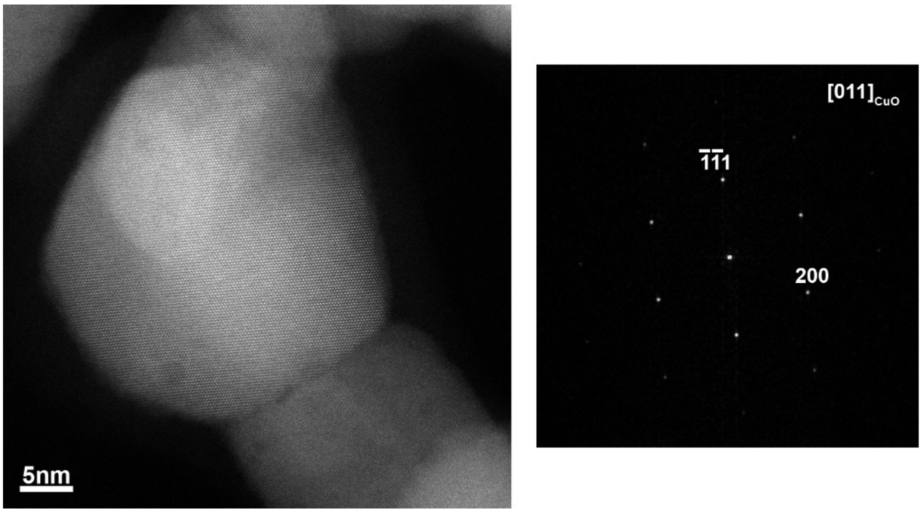

Figure S6. High-resolution HAADF-STEM image of CuO, together with the corresponding FT pattern.

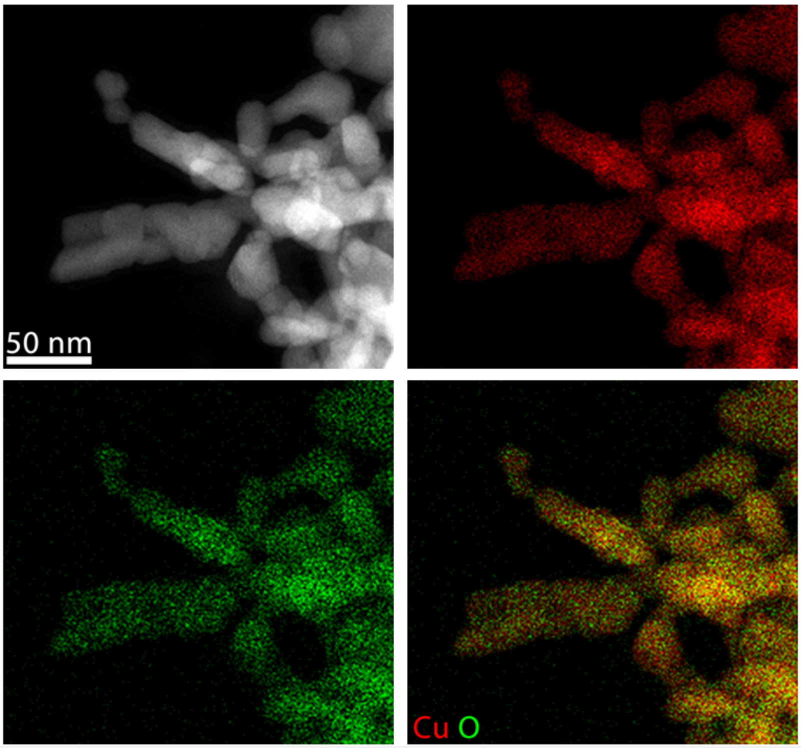

Figure S7. HAADF-STEM image of CuO particles and the corresponding EDS elemental maps of Cu and O, showing the distribution of the elements.

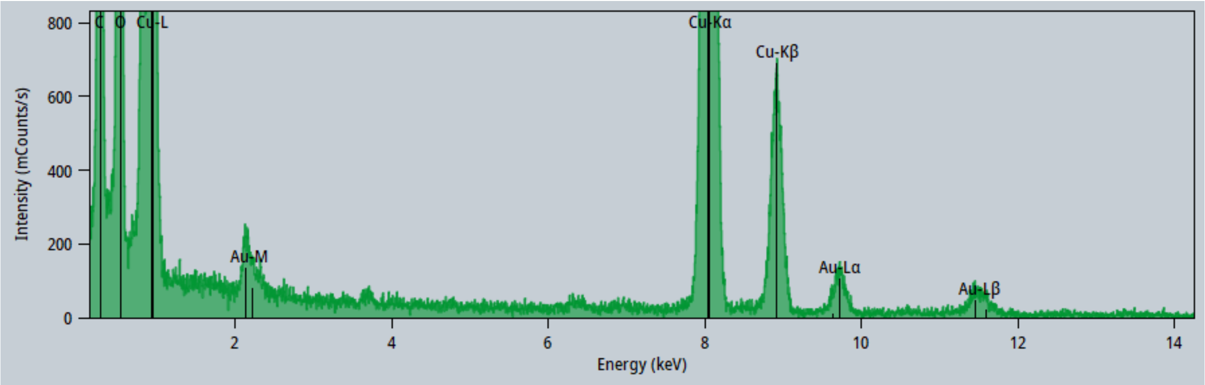

Figure S8. EDS spectrum acquired from the region shown in figure S7. The signal of Au originates from the Au TEM grid we used to deposit our sample.

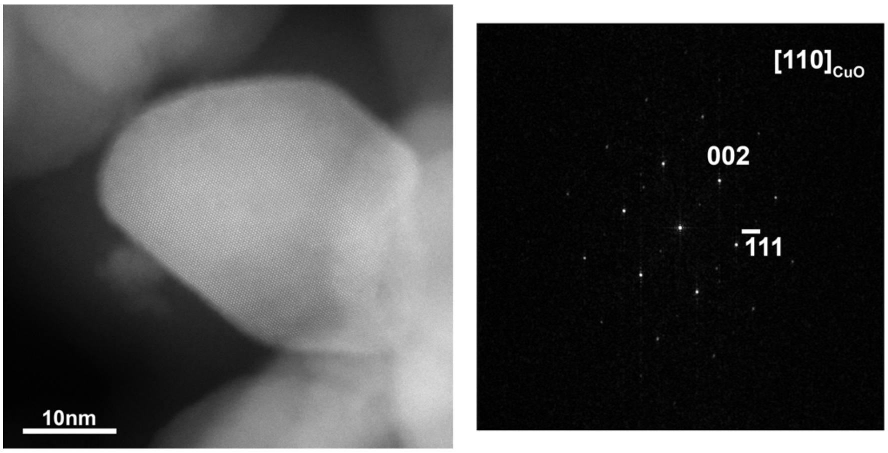

Figure S9. High-resolution HAADF-STEM image of the CuO-0.4%Sn sample, together with the corresponding FT pattern.

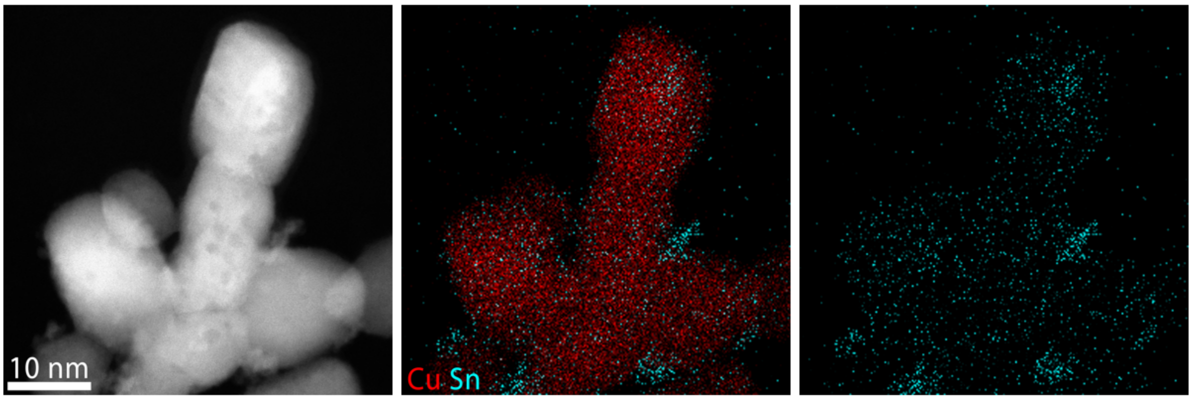

Figure S10. HAADF-STEM image of the CuO-0.4%Sn sample and the corresponding EDS elemental maps of Cu and Sn, showing the distribution of the elements.

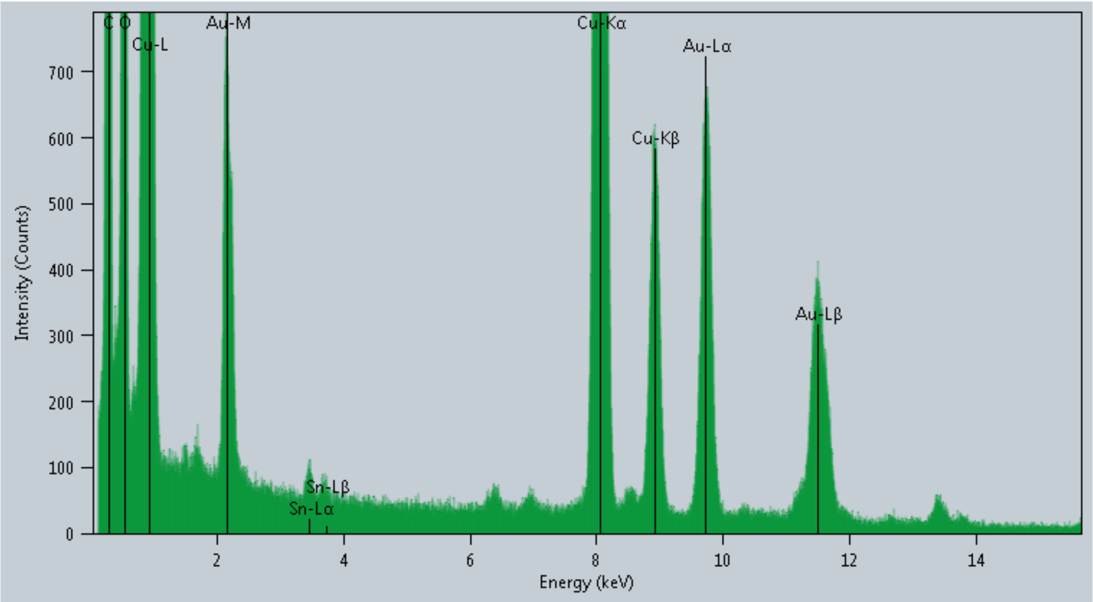

Figure S11. EDS spectrum acquired from the region shown in figure S10. A peak of Sn can be clearly observed. The signal of Au originates from the Au TEM grid we used to deposit our sample.

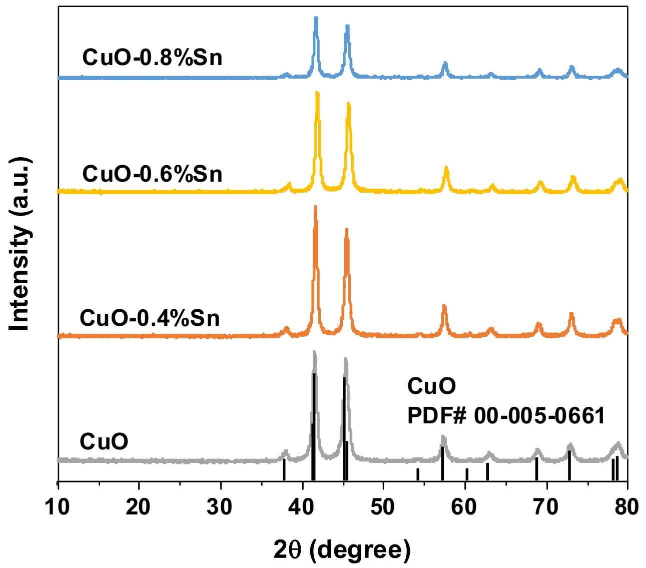

Figure S12. Comparison of the XRD patterns of CuO and CuO doped with different amounts of Sn (Co Kα1 1.78896 Å).

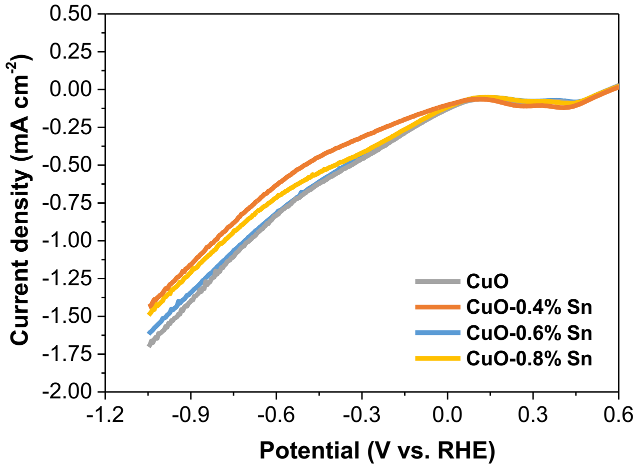

Figure S13. LSV curves of CuO, CuO-0.4%Sn, CuO-0.6%Sn and CuO-0.8%Sn in 0.1 M $KHCO_3$ electrolyte saturated with N_2 with a scan rate of 50 mV s^{-1}.

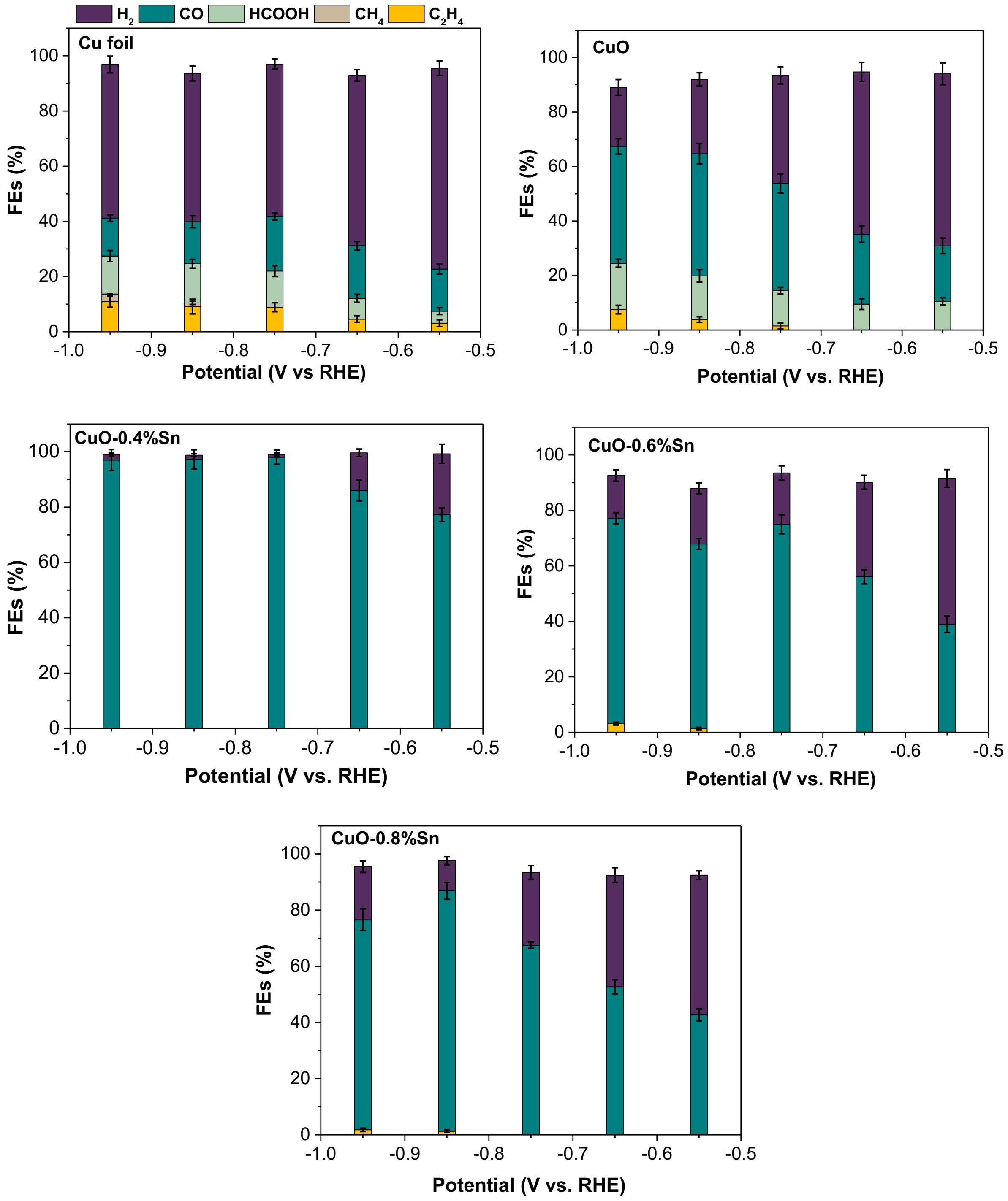

Figure S14. *i*R-corrected potential-dependent FEs of Cu foil, CuO, CuO-0.4%Sn, CuO-0.6%Sn and CuO-0.8%Sn. The error bars were obtained by performing the experiments in triplo.

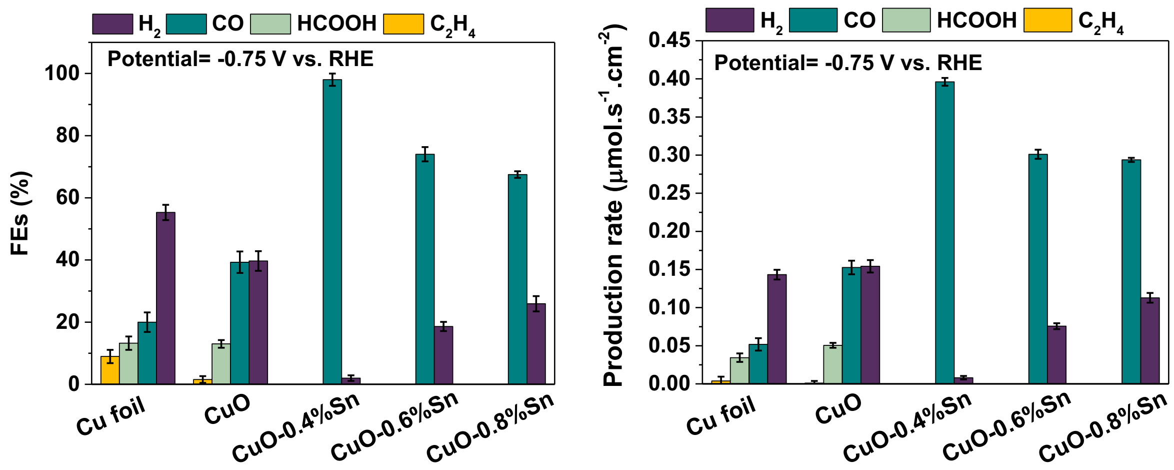

Figure S15. Comparation of FEs (left) and production rates (right) of Cu foil, CuO, CuO-0.4%Sn, CuO-0.6%Sn and CuO-0.8%Sn. The error bars were obtained by performing the experiments in triplo.

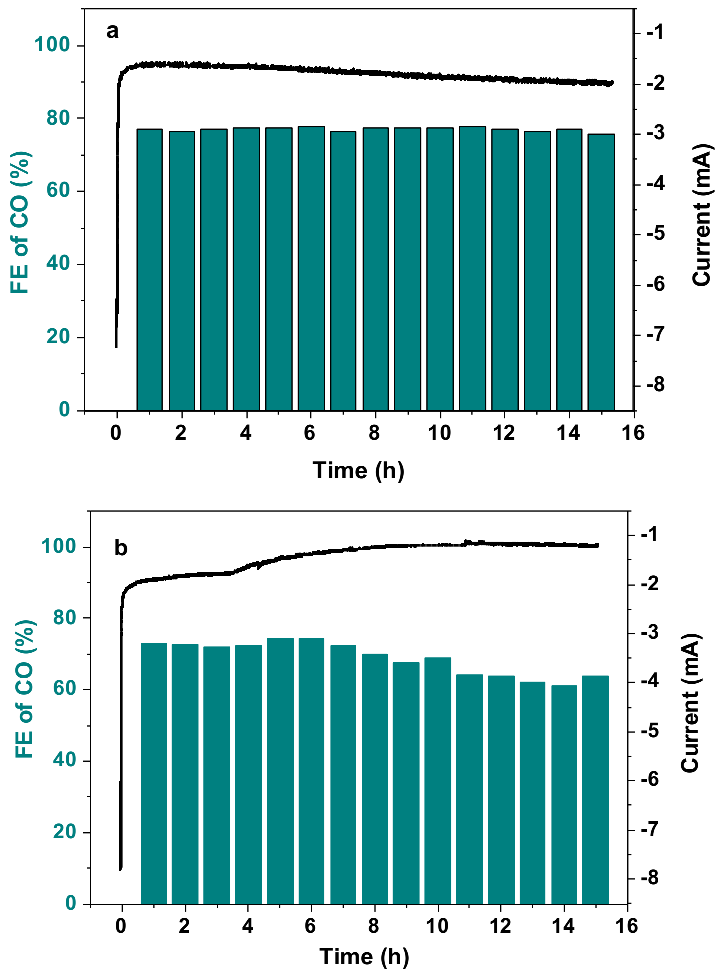

Figure S16. Stability of CuO-0.6%Sn (a) and CuO-0.8%Sn (b) at –0.75 V vs. RHE. The line and green column represent the i-t curve and the FE of CO, respectively. The decrease in current of CuO-0.8%Sn was observed, which could be attributed to the slight catalyst detachment during the long-lasted measurement.

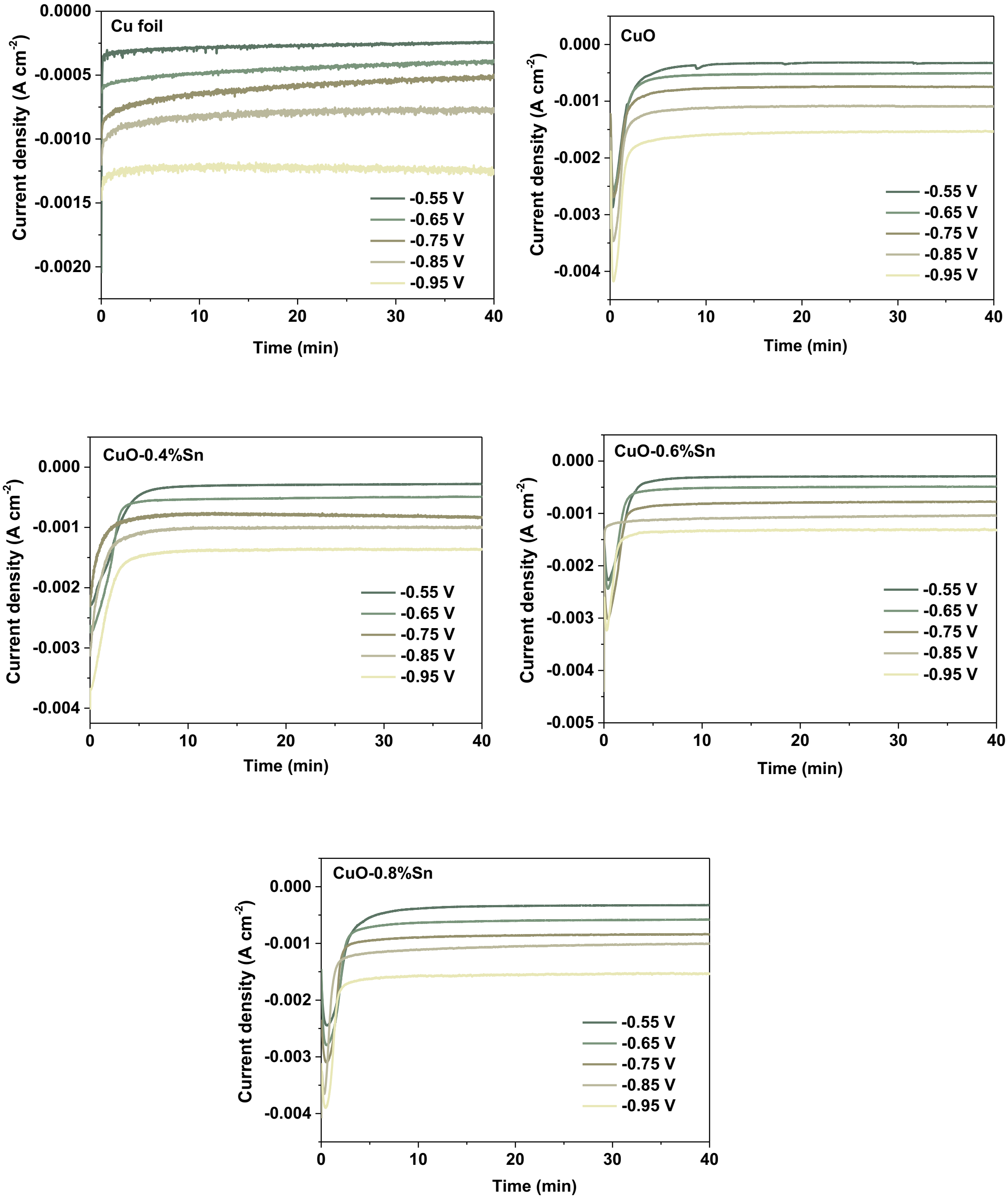

Figure S17. i-t curves of Cu foil, CuO, CuO-0.4%Sn, CuO-0.6%Sn and CuO-0.8%Sn at different potentials (V vs. RHE).

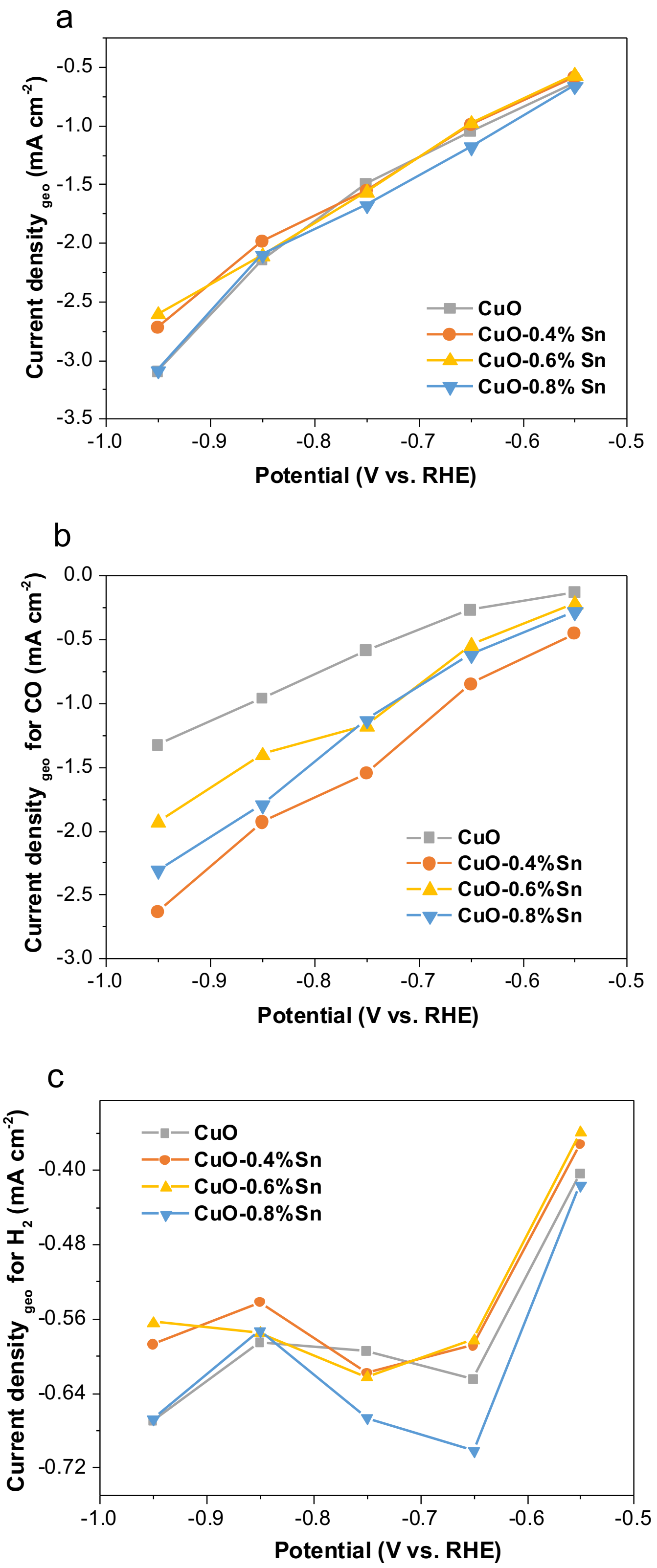

Figure S18. Geometric total current densities (a) and partial current densities of CO (b) and H_2 (c) in CuO, CuO-0.4%Sn, CuO-0.6%Sn and CuO-0.8%Sn at different potentials (V vs. RHE).

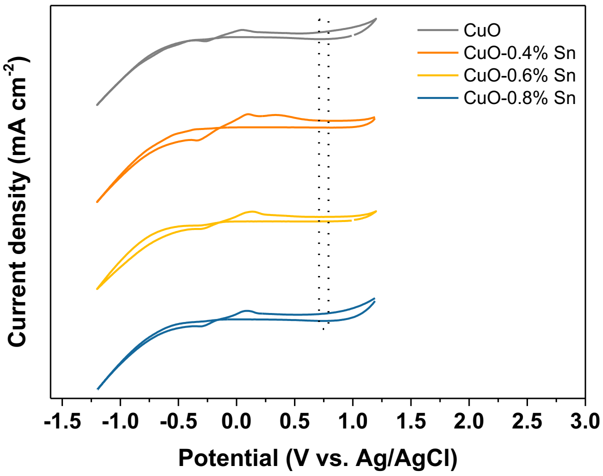

Figure S19. CV curves of the different Sn doped CuO samples at scan rate of 50 mV s^{-1} in CO_2 saturated 0.1 M $KHCO_3$ solution.

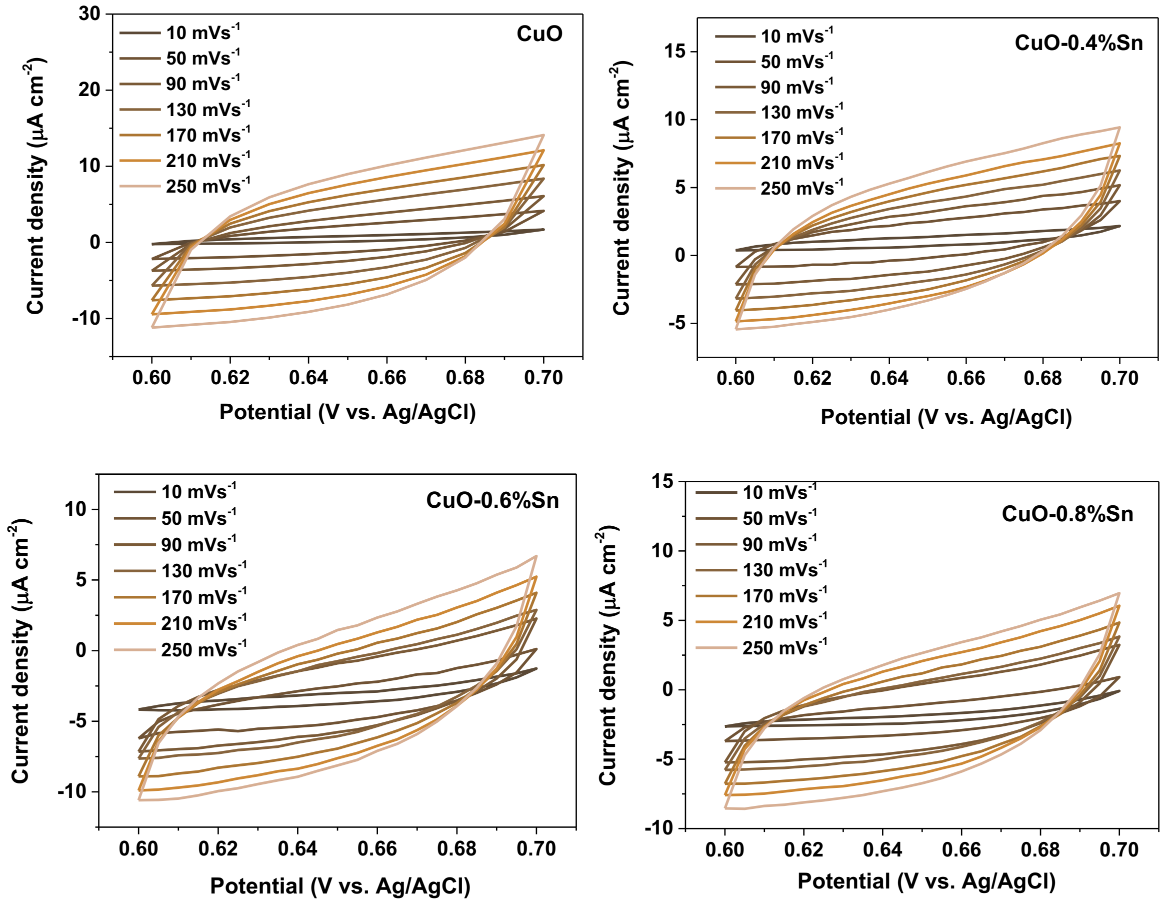

Figure S20. CV curves collected at different scan rates for ECSA measurements of CuO, CuO-0.4%Sn, CuO-0.6%Sn and CuO-0.8%Sn. The potential was chosen from the non-Faradaic region based on the full CV scan (Figure S19).

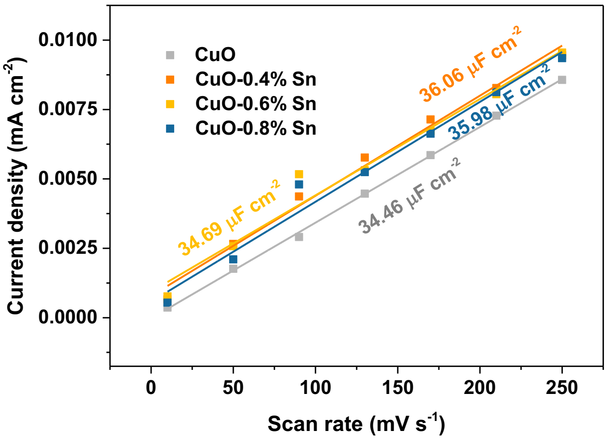

Figure S21. Charging current differences at 0.65 V vs. Ag/AgCl for all samples against scan rate for determining double-layer capacitance (C_{dl}).

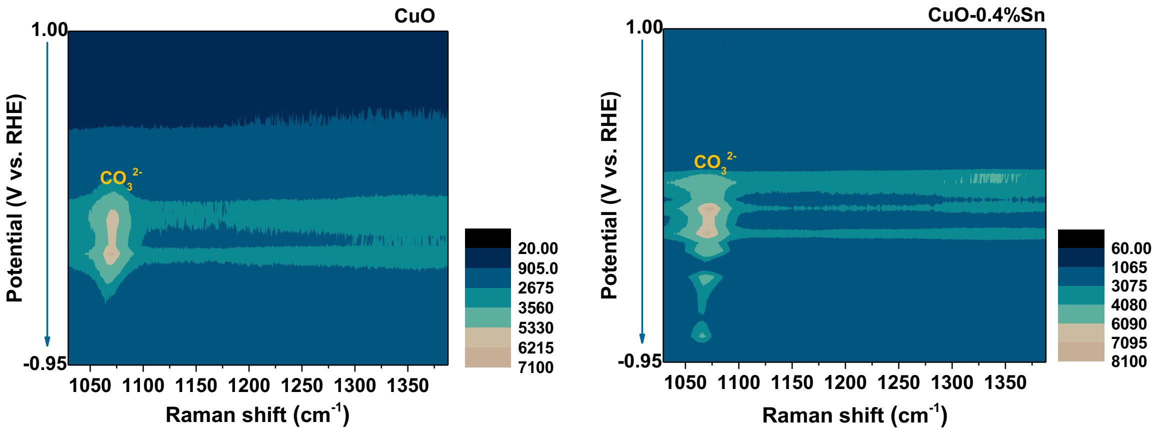

Figure S22. Potential-dependent *in situ* Raman spectra heatmap of CuO and CuO-0.4%Sn in the $*CO_3^{2-}$ region, with potentials scanning from 1.0 V to -0.95 V vs. RHE in CO_2 saturated 0.1 M $KHCO_3$ solution.

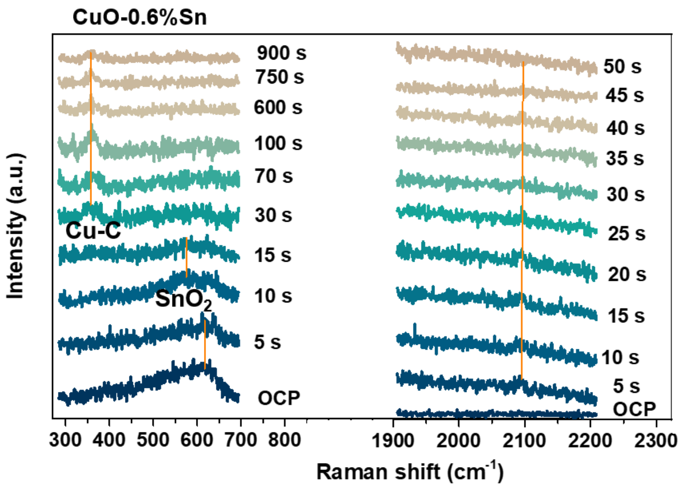

Figure S23. Time-dependent *in situ* Raman spectra of CuO-0.6%Sn in the oxide region (left) and the *CO region (right). The measurement was performed at -0.75 V vs. RHE in CO_2 saturated 0.1 M $KHCO_3$ solution.

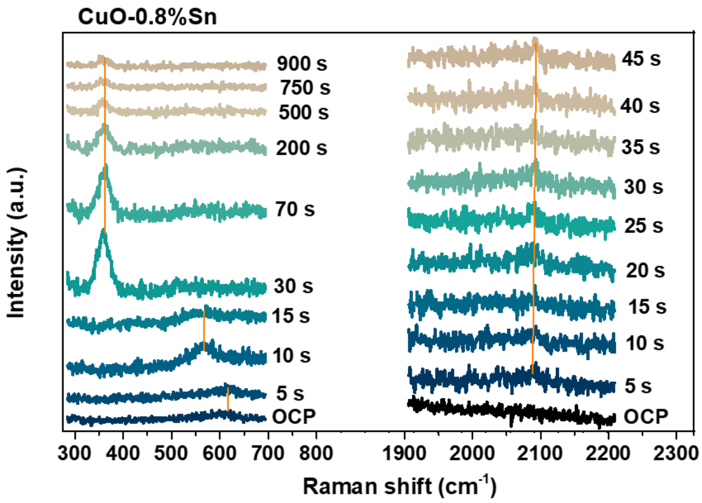

Figure S24. Time-dependent in situ Raman spectra of CuO-0.8%Sn in the oxide region (left) and the *CO region (right). The measurement was performed at -0.75 V vs. RHE in CO_2 saturated 0.1 M $KHCO_3$ solution.

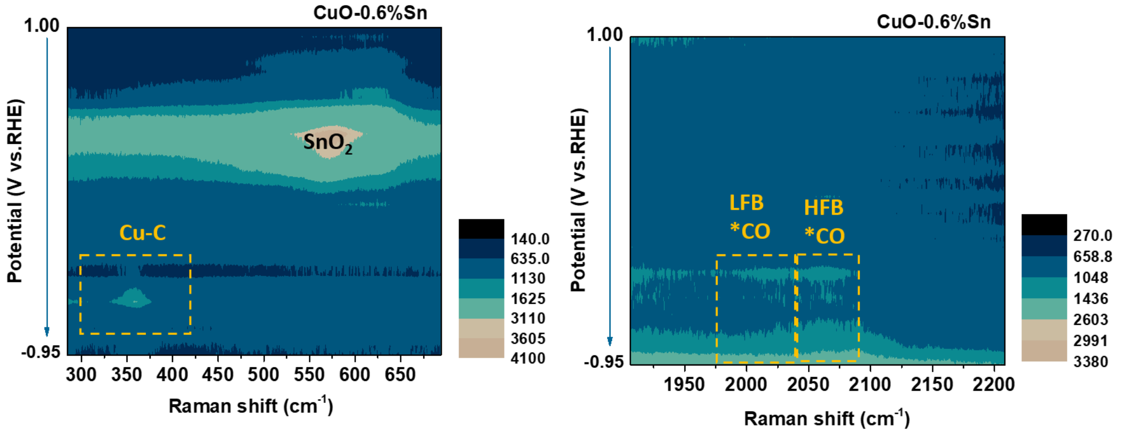

Figure S25. Potential-dependent *in situ* Raman spectra of CuO-0.6%Sn in the oxide region (left) and the *CO region (right), with potentials scanning from 1.0 to -0.95 V vs. RHE in a CO_2 saturated 0.1 M $KHCO_3$ aqueous electrolyte solution.

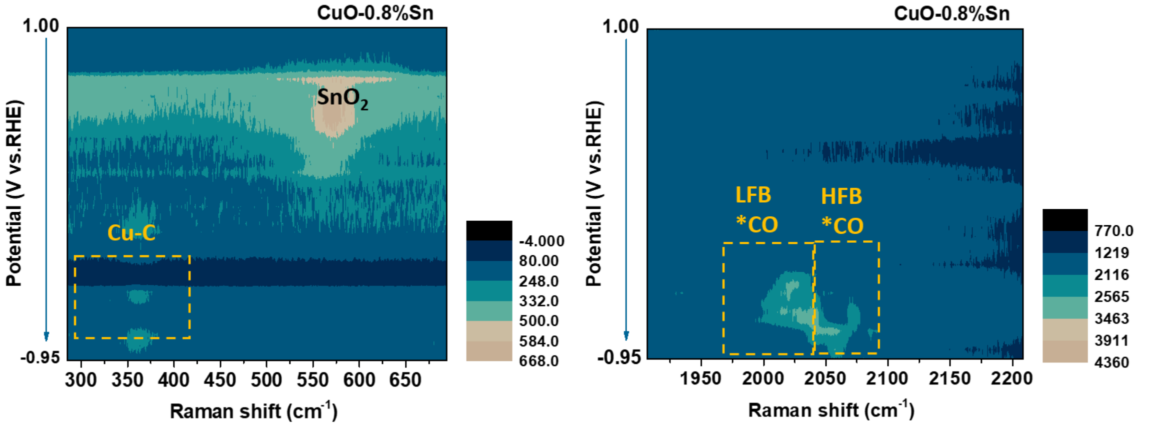

Figure S26. Potential-dependent *in situ* Raman spectra of CuO-0.8%Sn in the oxide region (left) and the *CO region (right), with potentials scanning from 1.0 to -0.95 V vs. RHE in CO_2 saturated 0.1 M $KHCO_3$ solution.

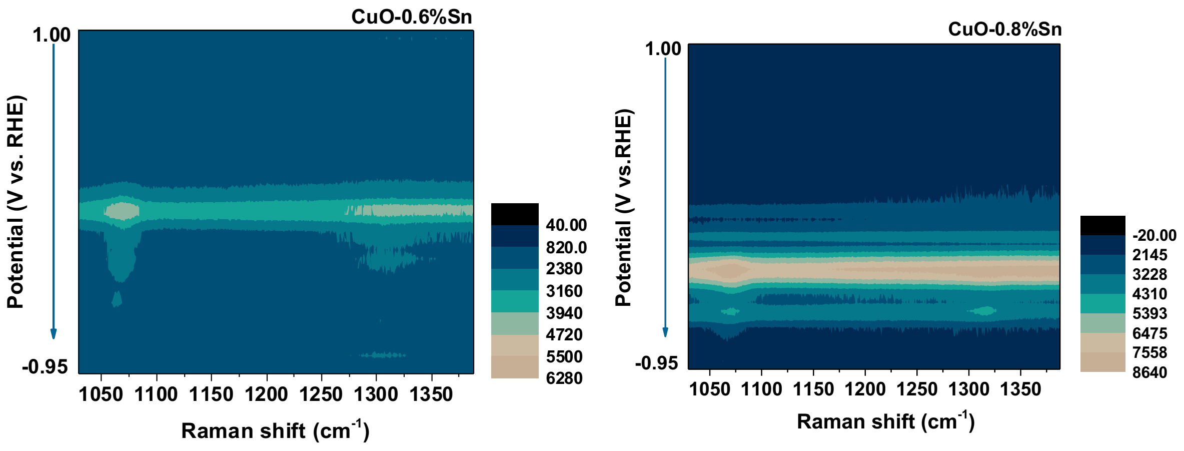

Figure S27. Potential-dependent *in situ* Raman spectra heatmap of CuO-0.6%Sn and CuO-0.8%Sn in the $^*CO_3^{2-}$ region, with potentials scanning from 1.0 to -0.95 V vs. RHE in CO_2 saturated 0.1 M $KHCO_3$ solution.

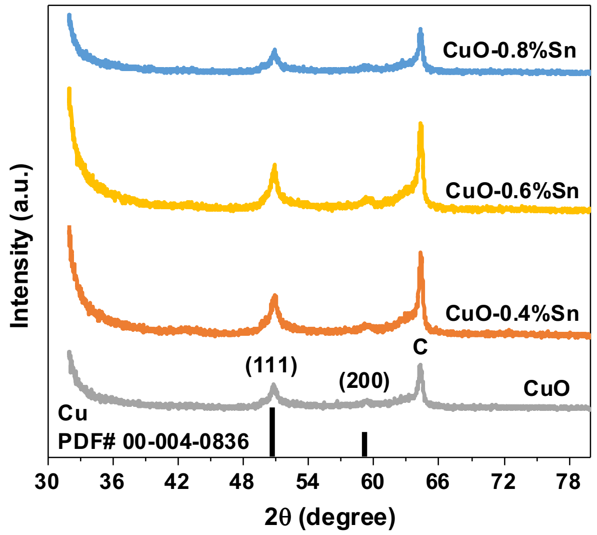

Figure S28. XRD patterns after electrochemical CO_2 reduction reaction (eCO_2RR) catalysis of CuO and CuO doped by different amounts of Sn (Co Kα1 1.78896 Å).

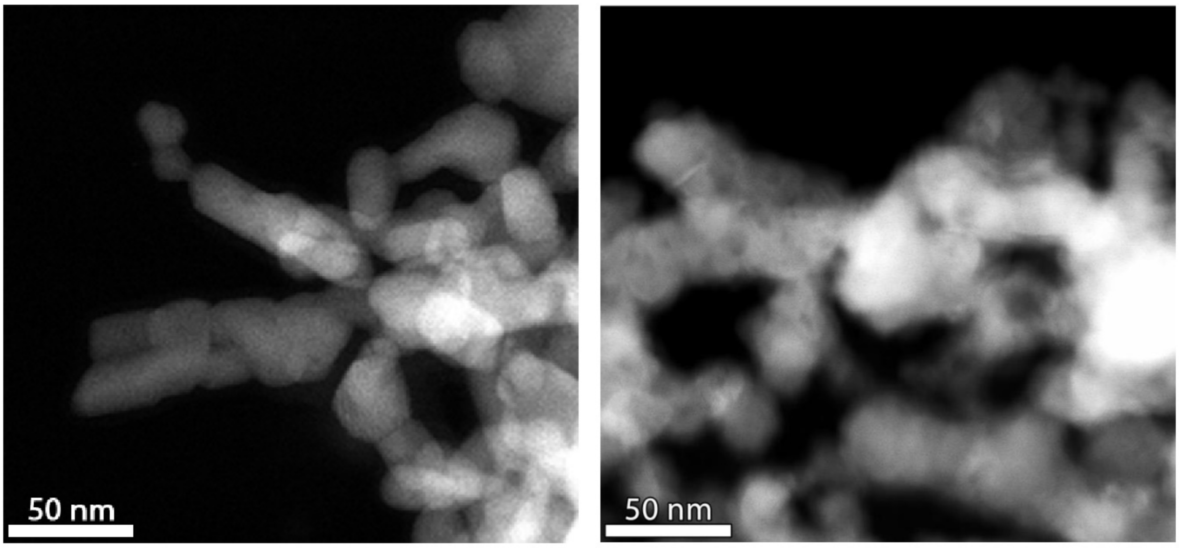

Figure S29. HAADF-STEM overview images of CuO before (left) and after eCO_2RR (right) catalysis. The sample was tested at –0.75 V vs. RHE for 40 min prior to ex situ HAADF-STEM analysis.

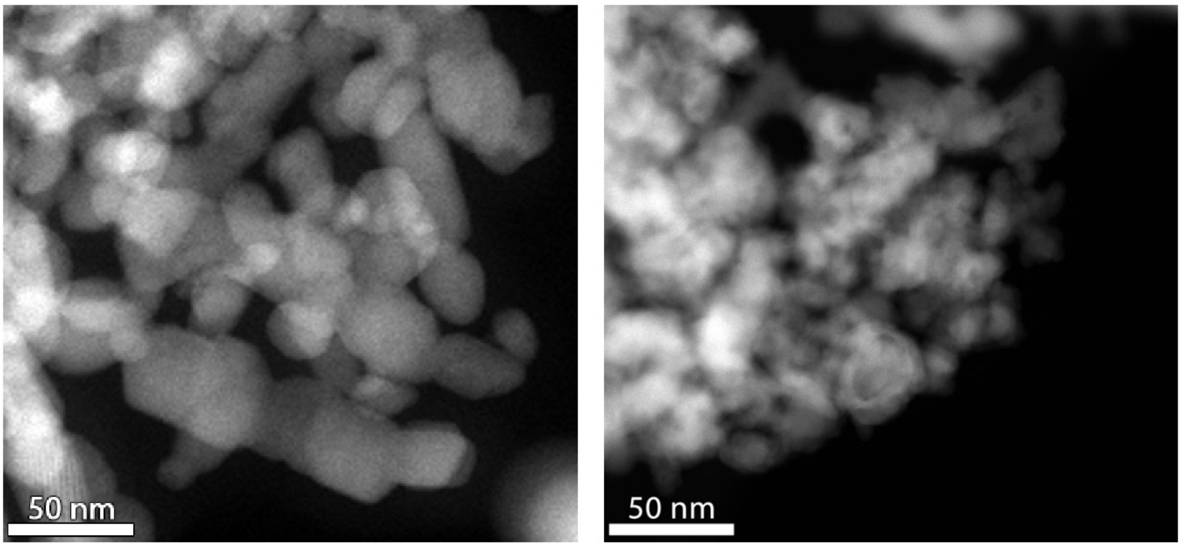

Figure S30. HAADF-STEM overview images of CuO-0.4%Sn before (left) and after eCO_2RR (right) catalysis. The sample was tested at –0.75 V vs. RHE for 40 min prior to ex situ HAADF-STEM analysis.

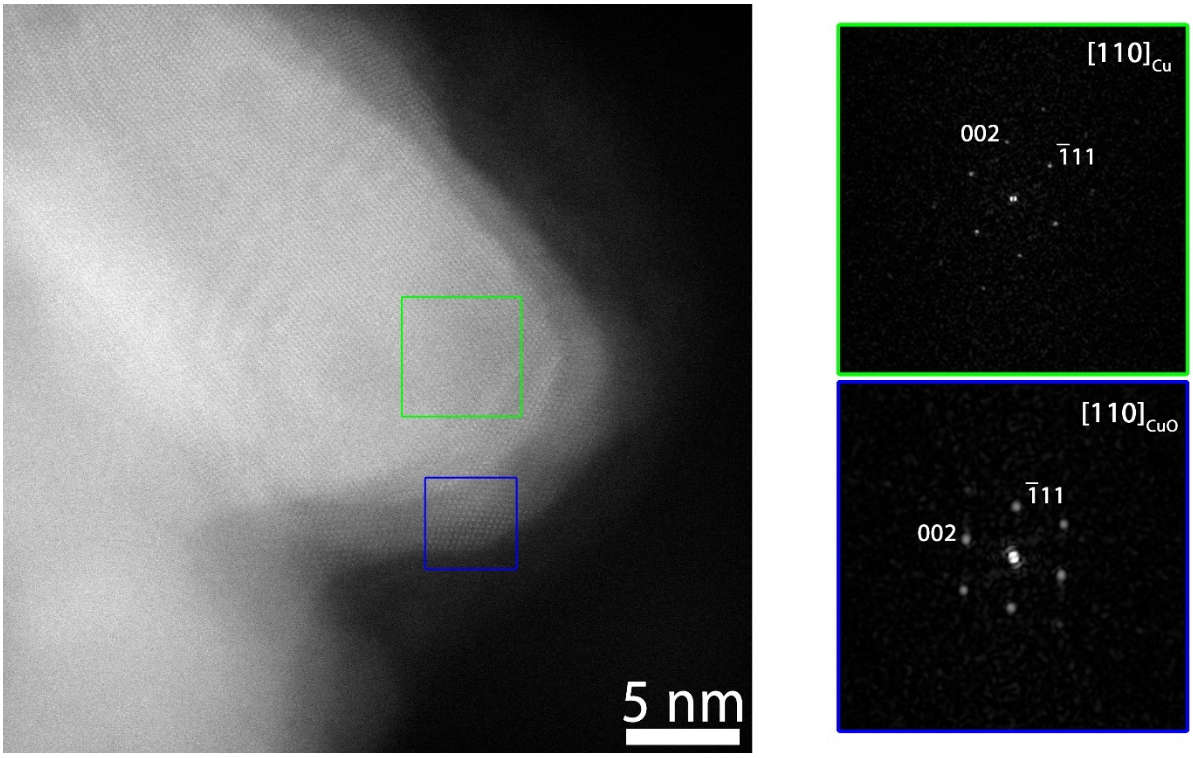

Figure S31. HAADF-STEM image of the CuO-0.4%Sn sample after eCO_2RR catalysis and the corresponding FT patterns from the regions indicated by the green and blue rectangles respectively. The CuO found on the outer layer is attributed to the inevitable oxidation during sample transfer. The sample was tested at –0.75 V vs. RHE for 40 min prior to ex situ HAADF-STEM analysis.

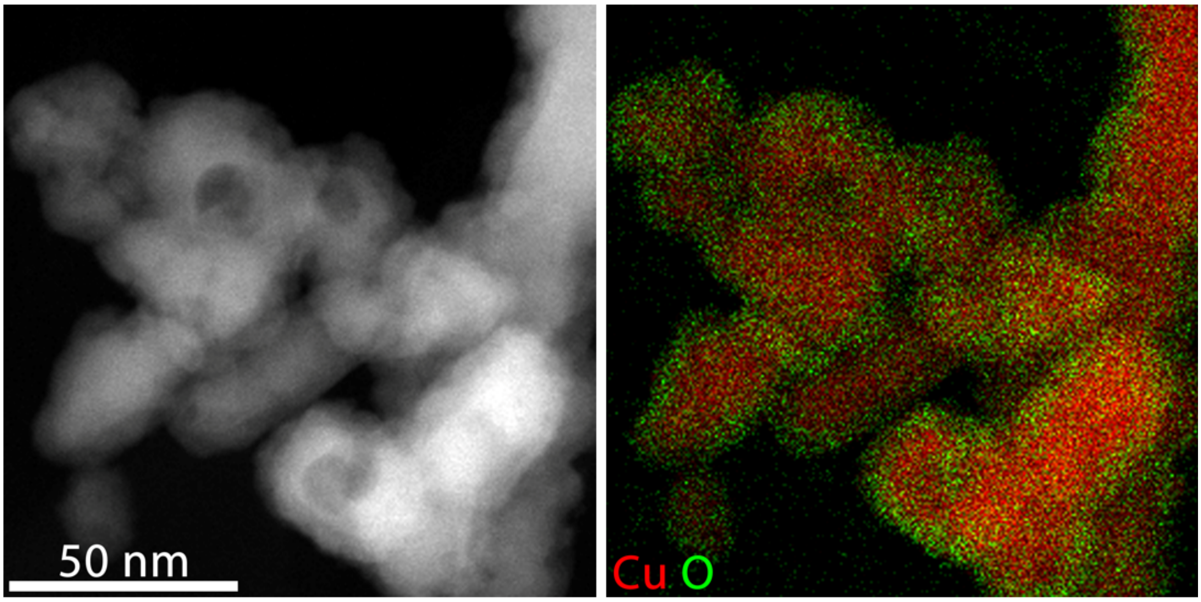

Figure S32. HAADF-STEM image of the CuO-0.4%Sn sample after eCO_2RR catalysis (left) and the corresponding EDS elemental maps (right) of Cu and O, showing the distribution of the elements. The sample was tested at –0.75 V vs. RHE for 40 min prior to ex situ HAADF-STEM analysis.

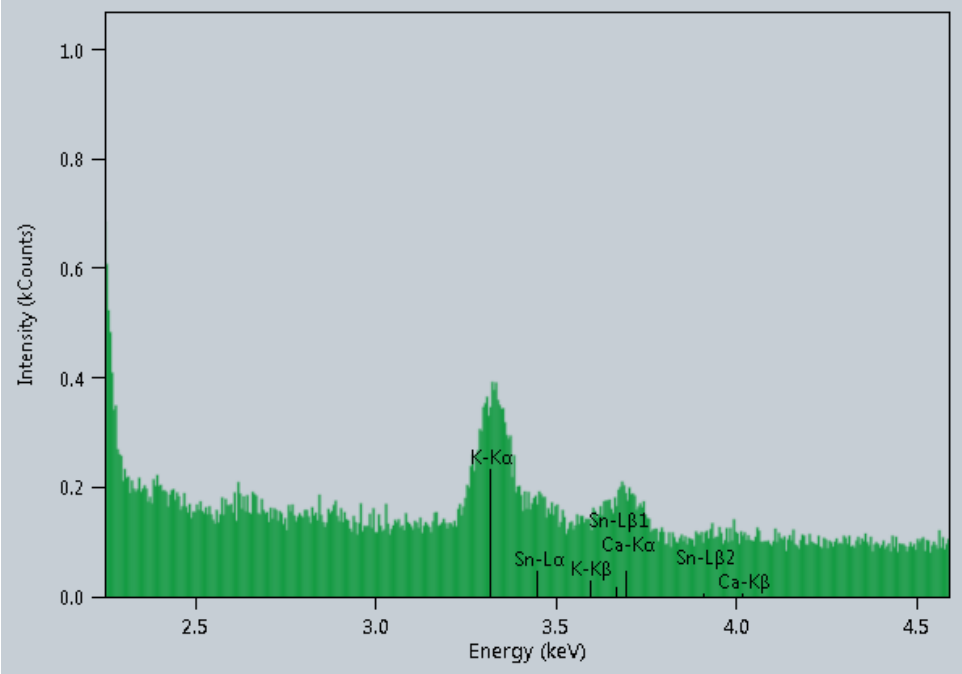

Figure S33. Magnified part of the EDS spectrum acquired from the region shown in Figure S32, where the L peak of Sn is expected. The signal of Sn is almost negligible and only K and Ca are present.

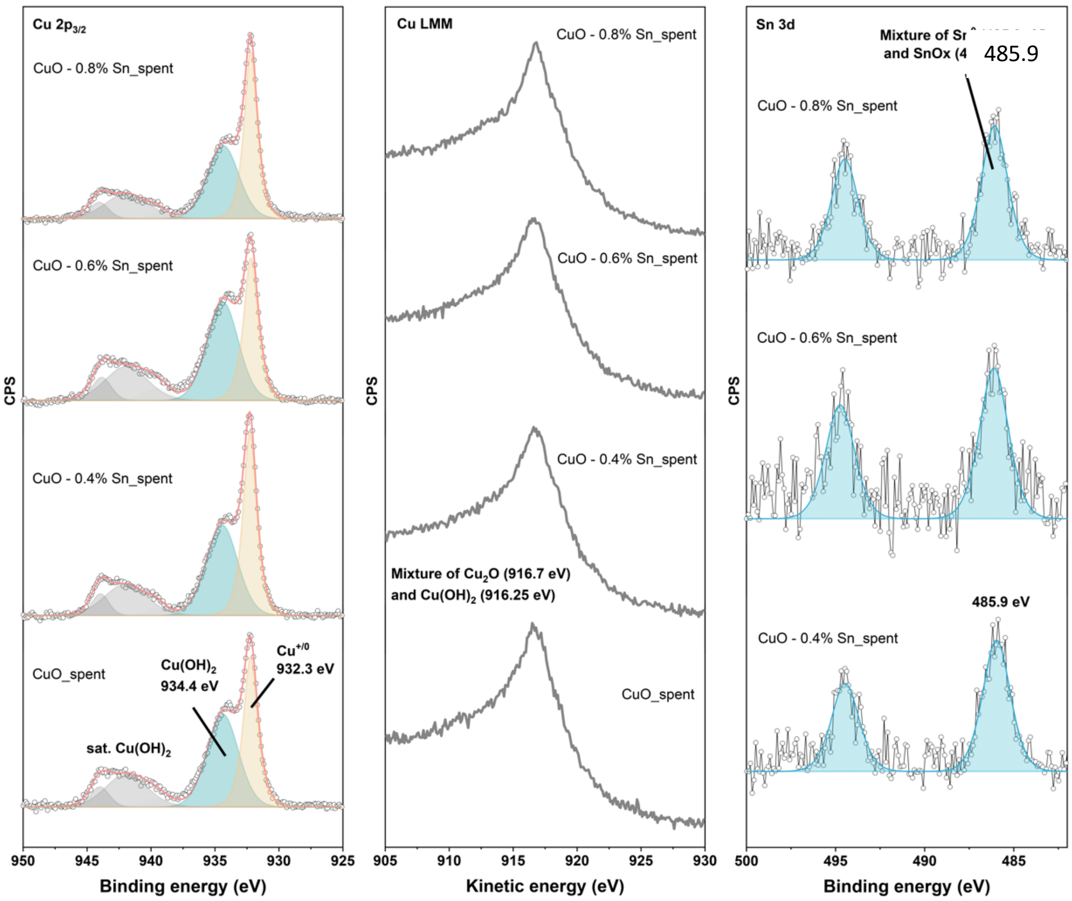

Figure S34. *Ex situ* XPS measurements on spent samples (CuO, CuO-0.4%Sn, CuO-0.6%Sn, CuO-0.8%Sn) at Cu 2p3/2 (left), Cu LMM (middle), and Sn 3d (right). All samples were tested at –0.75 V vs. RHE for 40 min prior to *ex situ* XPS analysis.

Supporting Discussion

The analysis of the spent samples by XPS reveals that the catalysts were reduced during the reaction but suffered from reoxidation due to air exposure. According to the analysis of the $Cu2p_{3/2}$ lines, it is clear that all materials are composed of a mixture of $Cu(OH)_2$ and $Cu^{+/0}$, located at 934.4 eV (blue) and 932.3 eV (orange) respectively. The presence of a $Cu(OH)_2$-like satellite (grey) confirms the presence of this specie. The ratios between these two contributions can be found in Table S6. The Cu LMM Auger region shows a maximum at 916.7 eV (Kinetic energy) for all materials. This agrees with the presence of Cu_2O and $Cu(OH)_2$ which were both reported at 916.7 eV and 916.25 eV respectively.[9] We confirm also the absence of Cu metal at the surface as no Cu^0 characteristic lines are visible in the LMM spectra. At last, the study of Sn 3d lines demonstrates that the Sn phase underwent reduction during the reaction but reoxidized in air as well. Compared to fresh samples (Fig. S4), the center of the lines shifted towards lower binding energies (485.9 eV) which indicates the reduction of the material. The surface is most likely composed of a mixture of SnOx and Sn^0, however, the low signal does not allow us to fit these two contributions with certainty. The Sn %at and Cu %at were calculated (Table S5). Compared to the fresh samples (Table S2), it is clear that the content of Sn on the surface of the catalysts changed. The higher the Sn content was, the larger the decrease at the surface. Furthermore,

it is observed that some parts of the sample get Sn enriched while others are depleted. This indicates that Sn redistributes over the activated catalyst surface.

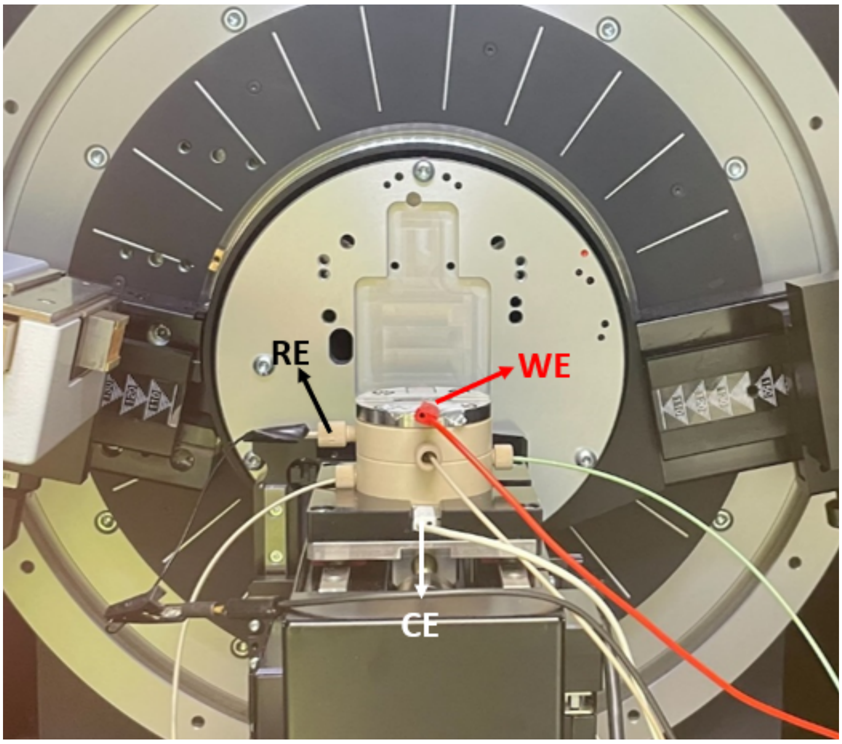

Figure S35. Picture of the custom-made *in situ* XRD cell used in this work.

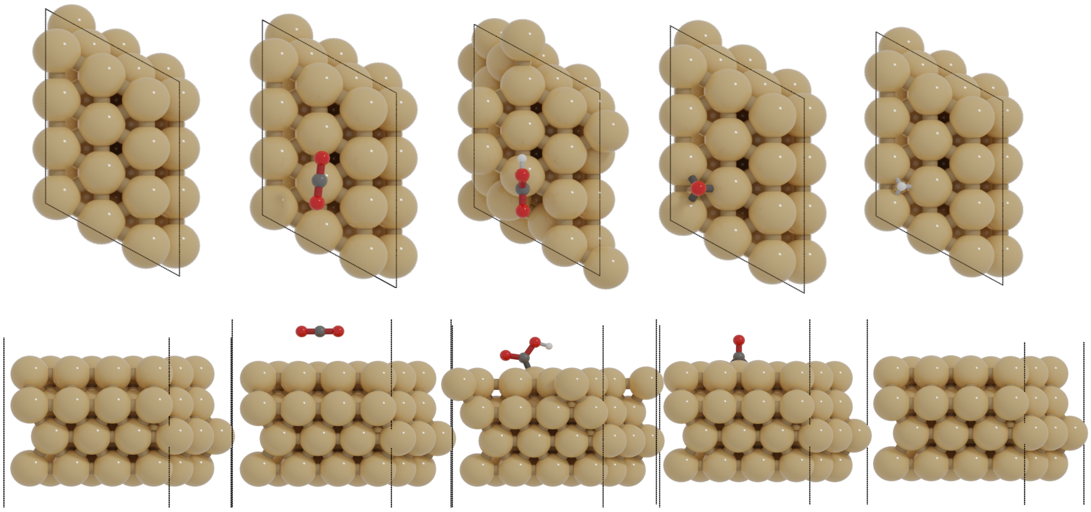

Figure S36. Top view and side view for the corresponding geometries of eCO_2RR on a Cu (111) model. Color code: yellow, Cu; dark grey, C; white, H; red, O.

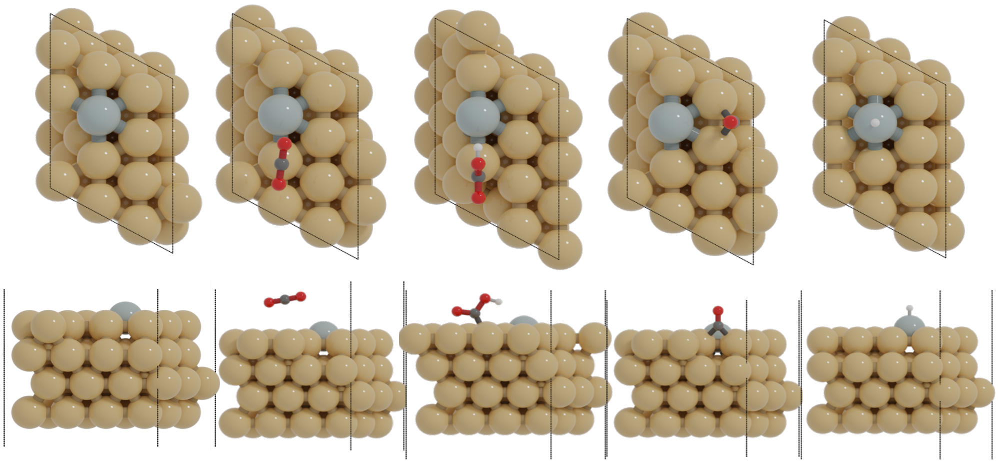

Figure S37. Top view and side view for the corresponding geometries of eCO_2RR on a $Cu_{x-1}Sn$ (111) model. Color code: yellow, Cu; light grey, Sn; dark grey, C; white, H; red, O.

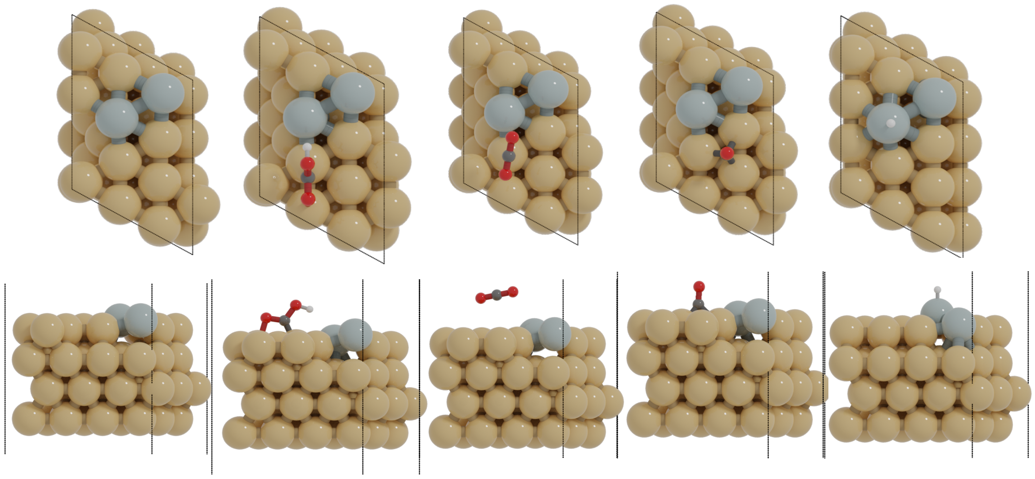

Figure S38. Top view and side view for the corresponding geometries of eCO_2RR on a $Cu_{x-2}Sn_2$ (111) model. Color code: yellow, Cu; light grey, Sn; dark grey, C; white, H; red, O.

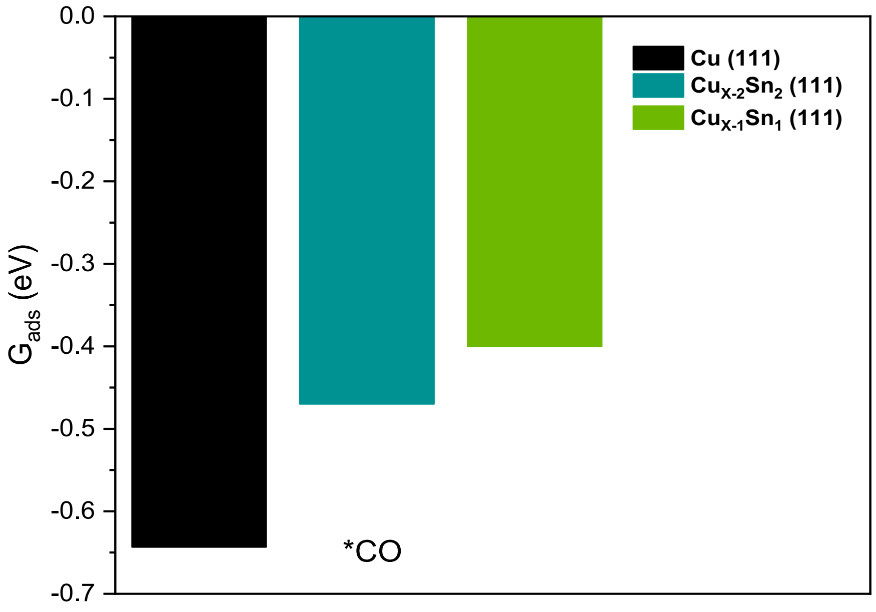

Figure S39. Adsorption energy of CO specie on various surface models.

D. References

(1) Wang, W. Z.; Wang, G. H.; Wang, X. S.; Zhan, Y. K.; Liu, Y. K.; Zheng, C. L. Synthesis and Characterization of Cu_2O Nanowires by a Novel Reduction Route. *Adv. Mater.* **2002**, *14*, 67-69.

(2) Kresse, G.; Furthmuller, J.; Hafner, J. Theory of the Crystal Structures of Selenium and Tellurium: The Effect of Generalized-Gradient Corrections to the Local-Density Approximation. *Phys. Rev. B* **1994**, *50*, 13181.

(3) Blochl, P. E. Projector Augmented Rave Method. *Phys. Rev. B* **1994**, *50*, 17953.

(4) Perdew, J. P.; Burke, K.; Ernzerhof, M. Generalized Gradient Approximation Made Simple. *Phys. Rev. Lett.* **1996**, *77*, 3865.

(5) Grimme, S.; Antony, J.; Ehrlich, S.; Krieg, H. A Consistent and Accurate Ab Initio Parametrization of Density Functional Dispersion Correction (DFT-D) for the 94 Elements H-Pu. *J. Chem. Phys.* **2010**, *132*, 154104.

(6) Mathew, K.; Sundararaman, R.; Letchworth-Weaver, K.; Arias, T. A.; Hennig, R. G. Implicit Solvation Model for Density-Functional Study of Nanocrystal Surfaces and Reaction Pathways. *J. Chem. Phys.* **2014**, *140*, 084106.

(7) Nørskov, J. K.; Rossmeisl, J.; Logadottir, A.; Lindqvist, L.; Kitchin, J. R.; Bligaard, T.; Jónsson, H. Origin of the Overpotential for Oxygen Reduction at a Fuel-Cell Cathode. *J. Phys. Chem. B* **2004**, *108,* 17886–17892.

(8) Tobin, J. P.; Hirschwald, W.; Chunningham, J. XPS and XAES studies of transient enhancement of Cu^1 at CuO surfaces during vacuum outgassing. *Appl. Surf. Sci.* **1983**, *16*, 441-452.

(9) Biesinger M., *Surf. Interface Anal.* **2017**, *49*, 1325-1334.
